# Pre-Pulse Inhibition of an escape response in adult fruit fly, *Drosophila melanogaster*

**DOI:** 10.1038/s41398-025-03717-5

**Published:** 2026-01-08

**Authors:** Erika Viragh, Lenke Asztalos, Michaela Fenckova, Tamas Szlanka, Zoltan Gyorgypal, Karoly Kovacs, Joanna IntHout, Pavel Cizek, Mihaly Konda, Emanuela Konda-Szucs, Agnes Zvara, Judit Biro, Eniko Csapo, Tamas Lukacsovich, Gabor Steinbach, Zoltan Hegedus, Laszlo Puskas, Annette Schenck, Zoltan Asztalos

**Affiliations:** 1 Institute of Biochemistry, HUN-REN Biological Research Centre, Szeged, Hungary; 2 Aktogen Hungary Ltd., Szeged, Hungary; 3 Aktogen Ltd., Department of Genetics, University of Cambridge & Ramsey, Huntingdon, Cambridge, United Kingdom; 4 Department of Human Genetics, Donders Institute for Brain, Cognition and Behaviour, Radboud University Medical Center, Nijmegen, the Netherlands; 5 Department of Molecular Biology and Genetics, Faculty of Science, University of South Bohemia in Ceske Budejovice, Ceske Budejovice, Czech Republic; 6 Bioinformatics Laboratory, Core Facility, HUN-REN Biological Research Centre, Szeged, Hungary; 7 HCEMM-BRC Metabolic Systems Biology Lab, Szeged, Hungary; 8 Department for Health Evidence (HEV), Radboud University Medical Center, Nijmegen, The Netherlands; 9 Voalaz Ltd., Szeged, Hungary; 10 Laboratory of Functional Genomics, Core Facility, HUN-REN Biological Research Centre, Szeged, Hungary; 11 University of Zurich, Brain Research Institute, Zurich, Switzerland; 12 Cellular Imaging Laboratory, Core Facility, HUN-REN Biological Research Centre, Szeged, Hungary; 13 Department of Biochemistry and Medical Chemistry, Medical School, University of Pecs, Pecs, Hungary

**Keywords:** Predictive markers, Schizophrenia, Molecular neuroscience

## Abstract

Pre-Pulse Inhibition (PPI) is a neural process where suppression of a startle response is elicited by preceding the startling stimulus (Pulse) with a weak, non-startling one (Pre-Pulse). Defective PPI is widely employed as a behavioural endophenotype in humans and mammalian disorder-relevant models for neuropsychiatric disorders. We have developed a user-friendly, semi-automated, high-throughput-compatible *Drosophila* light-off jump response PPI paradigm, with which we demonstrate that PPI, with similar parameters measured in mammals, exists in adults of this model organism. We report that *Drosophila* PPI is affected by reduced expression of *Dysbindin* and both reduced and increased expression of *Nmdar1* (N-methyl-D-aspartate receptor 1), perturbations associated with schizophrenia. Studying the biology of PPI in an organism that offers an abundance of genetic tools and a complex and well characterized connectome will greatly facilitate our efforts to gain deeper insight into the aetiology of human mental disorders, while reducing the need for mammalian models.

An estimated one in three people report mental health problems at some point in their lives. This makes mental disorders one of the biggest health burdens on individuals, families and societies [1]. The employment of biological model systems in basic science and pre-clinical studies for treatment development is inevitable. As there are no reliable biochemical or cellular tests for mental disorders, these model systems must include intact animals [2].

It is important to establish common intermediate phenotypes or biomarkers, in genetic epidemiology and psychiatric genetics commonly referred to as endophenotypes, to create a link between the experimental measures in animal models and human individuals. Pre-Pulse Inhibition (PPI) is a neural process where suppression of a startle response is elicited by preceding the startling stimulus (Pulse) with a weak, non-startling one (Pre-Pulse). PPI is considered to be an operational measure of sensorimotor gating, or the ability of a sensory event to modulate a motor response [3]. Measuring PPI is used to identify deficits in early-stage information processing. Impaired PPI is primarily reported in schizophrenic individuals but also in individuals with other psychiatric disorders [4]. PPI performance is likely an indicator of basic aspects of inhibitory neural processes. PPI and similar indicators are amenable to be studied in cross-species models relevant to these disorders. Abnormal PPI is extensively used as a behavioural endophenotype in humans and disorder-relevant mammalian models, and reveals neuropsychiatric disorder states and potential antipsychotic drug effects [4, 5].

Although results obtained from vertebrate models — most commonly the rat [6, 7] (*Rattus norvegicus domesticus*), mouse [8, 9] (*Mus musculus*), and zebrafish larvae [10, 11] (*Danio rerio*) — as well as invertebrate disease models, such as the locust *Locusta migratoria* [12] and the sea slug *Tritonia diomedea* [13, 14], have significantly enriched our knowledge, important questions in the field remain unanswered. Current research in sensorimotor gating focuses on elucidating the underlying mechanisms, including neural circuitry and genetic changes that contribute to impaired gating in various psychiatric disorders, and on exploring potential targeted interventions.

Research on the neural circuits involved in PPI — particularly the limbic cortex, striatum, pallidum, and pontine tegmentum (CSPP circuitry) — has advanced our understanding of sensorimotor gating problems in both animals and humans [6]. More recent studies have further expanded this knowledge by emphasizing the role of specific neurotransmitters, including glutamate, glycine, GABA, as well as brain regions such as the amygdala and caudal pontine reticular nucleus, in regulating PPI [7, 8]. Nevertheless, cellular-level circuit analysis remains a significant challenge in mammalian brains due to their complex cellular heterogeneity and connectivity.

*Drosophila* serves as a valuable complementary model, leveraging its simple, genetically tractable nervous system to uncover fundamental circuit mechanisms that can inform studies of more complex mammalian systems. In *Drosophila* a very sophisticated set of genetic tools are available and continue to expand. These tools include connectome databases [9–11], genetic methods for generating targeted mutants [12, 13] and for neural circuit-specific manipulation [14, 15], moreover neural activity detection and optogenetic toolkits [16].

Approximately 600 genes have been implicated in schizophrenia susceptibility [17]_._ Comparative genomics studies indicate that a substantial proportion of these genes — up to 81% of 344 risk genes — have orthologues in the fruit fly [18]. The presence of these orthologues, combined with *Drosophila*’s powerful genetic tools, rapid life cycle, and cost-effectiveness, makes it a valuable model for investigating the genetic, neurochemical, and behavioural aspects of schizophrenia.

Research in the fruit fly *Drosophila* has already contributed to uncovering biological mechanisms underlying psychiatric disorders [19–22]. The development of a high-throughput-compatible PPI paradigm for adults could significantly advance progress in this field [21].

The PPI phenomenon in *Drosophila* was first described in larva, using a startle response to the buzz of a wasp predator [23, 24]. Even though the behaviour paradigm is robust enough to show effects for RNA interference (RNAi) or mutations in genes associated with human mental disorders, it has not been adapted to perform experiments in high throughput.

It has been previously observed both at electrophysiological and behavioural levels that the adult *Drosophila* escape/startle response, the light-off jump response, can be habituated [25]. In these light-off jump response habituation behaviour experiments single *Drosophila* were observed by an experimenter, and the presence of jump responses was noted manually. To improve this system, we have developed a semi-automated method for the detection of *Drosophila* jump responses, which allows precise control of experimental variables and relatively high throughput. In this behaviour paradigm it is now feasible to conduct large-scale genetic or compound screens that affect habituation [26].

We report here the discovery that the light-off jump response can also be suppressed by a preceding dimming of light, expanding the applicability of this behaviour to measure Pre-Pulse Inhibition properties in adult *Drosophila*.

To validate our PPI paradigm in the neuropsychiatric field we tested *Drosophila* adults in which we manipulated the orthologues of two established human schizophrenia susceptibility genes. In our “schizophrenia models” *Nmdar1* [27–31] (*N-methyl-D-aspartate receptor 1*), an orthologue of *GRIN1* was either down- or up-regulated, while *Dysbindin* [32–35], an ortholog of *DTNBP1* was down-regulated.

NMDAR is one of three types of ionotropic glutamate receptors in vertebrate neurons and it has an important role in synaptic plasticity and learning & memory functions [36]. NMDA receptor defects can lead to nervous system disorders [37], and there is a well-supported “glutamate hypothesis” of schizophrenia [38, 39]. NMDA receptors exist in the *Drosophila* nervous system, as well, and play an important part in learning & memory processes [27, 28, 30, 31]. In accordance with this evolutionary conservation, we were able to detect decreased PPI performance both in knock-down and overexpression of *Drosophila Nmdar1* in our PPI system (see Results).

*Dysbindin-1* (dystrobrevin-binding protein 1, encoded by *DTNBP1*) was identified as a constituent of the BLOC-1 (biogenesis of lysosome-related organelles complex 1) and DPC (dystrophin-associated protein complex) complexes, which are involved in maintaining the structure of muscle as well as neuronal synaptic membrane and in endosomal trafficking, neurotransmitter release as well as neural development, respectively [40–44].

Mutations in the *DTNBP1* gene or reduction in its mRNA and protein levels are associated with a higher occurrence of schizophrenia [45–47]. Rodent and *Drosophila* models of *DTNBP1* defects successfully recapitulated some of the schizophrenia hallmarks and phenotypes, such as changes in neurotransmitter release and impaired social behaviour, as well as cognitive deficits, and contributed significantly to the understanding of schizophrenia aetiology [32–35, 47, 48]. In our new behaviour paradigm, knock-down of *Drosophila Dysbindin* was also characterized by PPI defects.

Based on these findings the use of the *Drosophila* PPI behavioural paradigm presented here permits experiments that up to date have been unfeasible in mammalian/rodent disorder-related models and are characterized by much lower cost and higher efficiency. The PPI paradigm established has the potential to fundamentally contribute to the understanding of molecular, genetic and neural processes underpinning mental capacities, their deficits in disorders and open up avenues to novel treatment strategies.

## Results

### Light-off jump response system with added PPI functionality

We have previously developed a semi-automated, high-throughput behaviour system to measure neural processes in the visual sensory domain of the fruit fly. Some of its fundamental properties — such as the specificity of jumps in response to light-off stimuli and the accurate coupling of jump detection to wing vibration — have been described [26].

### System details

Each fly is individually housed in a behaviour chamber, where its jump and flight responses are detected via wing vibration sounds. These responses are recorded in binary format (Yes/No, i.e., 1 or 0) using a pair of microphones positioned at both ends of the chamber. The system outputs data files that detail the occurrence and timing of jump responses triggered by light-off/dimming events (see flow chart in the Methods).

Eight behaviour chambers are built in a behaviour box, and two boxes comprise the automated, high-throughput light-off jump response Habituation and Pre-Pulse Inhibition system (Aktogen Limited; see Supplementary Fig. 1). Since one experimenter can operate two systems simultaneously, up to 32 flies can be tested in parallel.

The chambers, made of polytetrafluoroethylene, are illuminated with green light from 525 nm LED sources. Light-off stimuli are regulated by an Intelligent Light Controller Unit (ILC box) with an Arduino light controller board, providing high-precision control over both light intensity (ranging from 0–65, 535 arbitrary units) and duration (from 1–4, 294, 967, 295 ms). Light intensity in lux varies linearly with the arbitrary unit settings (see Supplementary Fig. 5).

To ensure accurate jump-and-flight response detection, each chamber is equipped with two microphones and a custom-built differential noise-cancelling amplifier. The noise-cancellation function electronically subtracts identical external noise signals detected by both microphones, enabling specific amplification of fruit-fly-generated sounds even in noisy environments (up to 50 dB). A 1000x linear sound amplification across the 1–880 Hz range, combined with an empirically defined noise threshold, ensures reliable detection of the startle response.

The amplified sound signals are collected and analysed using custom software developed in National Instruments LabVIEW. This software enables users to define experimental parameters or select from a range of optimised settings (e.g., stimulus intensity, duration, and inter-stimulus intervals).

**Fig. 1:**
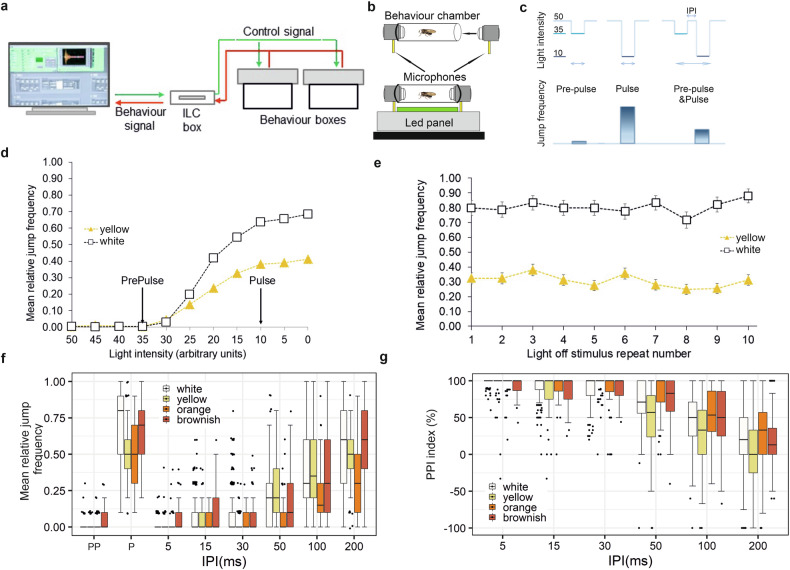
Light-off jump response system for measuring Pre-Pulse Inhibition in *Drosophila.* **a** Schematic representation of Light-off jump response system, (ILC: Intelligent Light Controller). **b** Each fly is tested individually in a behaviour chamber for its jump response, using microphones positioned at the ends of the chambers. **c** Elements of the Light-off jump response Pre-Pulse Inhibition protocol: weak [Pre-Pulse (PP), 50→35], strong [Pulse (P), 50→10] or combined weak-and-strong [PPI), 50→35 & 50→10] light dimming stimuli separated by Inter Pulse Interval (IPI). All light intensity values given here and subsequently are in arbitrary light units, except otherwise stated. **d** Jump response of white-eyed (*w*^*1118*^, n = 96) and yellow-eyed (*GMR-wIR*, n = 160) *Drosophila* to different light dimming stimuli (Initial Light Intensity 50, Dimmed light intensity range 50–0). “Pre-Pulse” and “Pulse” arrows represent the two chosen light intensity values used in subsequent PPI experiments. Each fly received 10 light-off or dimming stimuli. The number of jumps (“Yes” responses, scored as 1) was summed per fly to yield the jump frequency, then divided by 10 to calculate the Relative Jump Frequency. Data shown represent the Mean Relative Jump Frequency across all flies (n). **e** Repeats of light-off stimuli at 5 s ITI evokes non-habituating jump responses in white-eyed (n = 96) and yellow-eyed (n = 160) flies (50→0 light units, 15 ms duration). Light-off stimuli were repeated 10 times for the flies and the jump responses were averaged for each successive repeats (1–10 on X axis) for all flies. Experiments were conducted on three or five different days. **f** Pre-Pulse Inhibition at different IPI durations. Mean Relative Jump Frequencies of white-eyed (n = 95), yellow-eyed (n = 92), orange-eyed (n = 80) and brownish-eyed (n = 91) flies to PPI light dimming stimuli (described in **d**) at 5–200 ms IPIs are represented by box plots. Experiments were performed on three different days. **g** *Drosophila* PPI expressed in PPI indices. PPI indices are calculated from jump response data shown in panel **f** by the formula: 100 – ((PPI score/Pulse score) x100) [58]. For full genotypes see Supplementary Table 1.

We have now adapted this system to assess PPI (see Methods). The updated device (Fig. 1a, b; Supplementary Fig. 1) can precisely switch off and/or dim light in a fully automated manner to evoke the innate jump-and-flight startle response. The primary technical advancement is its ability to deliver complex “Pre-Pulse & Pulse” compound light stimuli, required for the PPI protocol (Fig. 1c).

### Selection of the Pulse and Pre-Pulse stimuli for PPI

The light-off jump response depends on the eye colour of the flies [49, 50]. Available genome-wide collections of *Drosophila* transgenic RNAi strains as well as mutants have a wide-range of eye colours, which would hamper large-scale screening and comparison of manipulated genotypes (Supplementary Fig. 3). For this reason, we aimed to find effective light-off/dimming stimuli used in PPI experiments for different eye-coloured flies.

When we exposed flies to a series of complete light-off stimuli starting from different Initial light-intensity values, strong jump responses were observed both in white-eyed (*w*^*1118*^) and in yellow-eyed flies (*GMR-wIR*, i.e., eye-specific *white* RNAi knock-down [51, 52]). Although the two strains show different maximum jump frequencies, they reach the “Initial Light Intensity vs. Jump response” curve’s asymptotes at very similar Initial Light Intensity values (Supplementary Fig. 4). Based on these results we chose 6.36 lux (corresponding to 50 arbitrary light units of the behaviour system, Supplementary Fig. 5) as default Initial Light Intensity for further light-off/dimming jump response experiments (see arrow in Supplementary Fig. 4). This Initial Light Intensity value evokes maximum response at the lowest possible light intensity so that overstimulating the visual system during the experiments could be avoided.

Next, we looked for an efficient Pre-Pulse stimulus that can inhibit the response to an appropriate Pulse stimulus in PPI experiments. When flies were exposed to light intensity reduction, i.e., dimming, jump responses still were observed. In dimming, Initial Light Intensity 50 was dimmed to X, i.e., “Dimmed light intensity”, where X > 0 (light dimming 50→X, Fig. 1d). From the resulting “Light-dimming vs. Jump response” curve, two X values for the dimming stimuli were selected, one for Pre-Pulse and one for Pulse. At 50→35 dimming, the flies produced infrequent jump responses, which showed that they were able to perceive the stimulus but the negligible response to it does not interfere with the Pre-Pulse Inhibition results. In PPI experiments we used this 50→35 dimming as Pre-Pulse stimulus. For Pulse we chose the stimulus 50→10 dimming, to which flies gave a strong jump response, comparable to the maximum response to a complete light-off stimulus (50→0), but potentially easier to suppress by the Pre-Pulse (Fig. 1d).

White-eyed flies showed stronger jump reactions than yellow-eyed flies to the same light-off stimuli (Fig. 1d, e; see Supplementary Table 1 for full genotype descriptions), whereas flies with wild-type red eye colour – consistent with previous reports [49, 50] – did not exhibit any jump responses to these light-off/dimming stimuli.

When complete light-off stimuli are presented frequently (e.g., every 1 s, i.e., 1 s Inter Trial Interval [ITI]), jump response habituation — i.e., a decrement in response due to repeated stimulus exposure — occurs (Supplementary Fig. 7a,b). This form of non-associative learning has been widely used in studies of cognitive disorders [26, 53–55]. In contrast, when light-off stimuli were presented less frequently, no influence of the previous stimulus on the response was observed (i.e., no response decrement), and at a 5 s ITI, subsequent trials can be considered independent (Fig. 1e; Supplementary Fig. 7c). Therefore, a 5 s ITI is suitable for repeated trials in PPI experiments.

Taken together, we established parameters for a light-off jump reflex Pre-Pulse Inhibition protocol in white- and yellow-eyed *Drosophila* (Fig. 1c). In this protocol, a weak Pre-Pulse dimming stimulus (50 → 35) is followed by a strong Pulse dimming stimulus (50 → 10), separated by various Inter Pulse Intervals (IPIs, to be determined). These compound PPI stimuli can be presented at 5 s Inter Trial Intervals (ITIs) in random order to elicit independent jump responses.

We conducted a series of additional experiments to determine whether flies with different eye colours could be induced to jump. We found that flies with various eye colours exhibited jump responses to increased Initial Light Intensity. The closer the eye colour was to the wild-type red, the higher the Initial Light Intensity required to elicit a jump response (Supplementary Fig. 6).

We performed similar experiments with orange-, brownish-, brownish red-, and red-eyed flies, applying the same logic to select potential Pre-Pulse and Pulse values as we had used for white- and yellow-eyed flies. The results are summarized in Supplementary Table 2 (see also Supplementary Fig. 3 and the Methods section for eye colour characterization).

### Light-off jump response PPI is observed in adult *Drosophila*

The Pre-Pulse and Pulse light-dimming stimuli and their temporal relationship is depicted in Fig. 1c. To establish optimal Inter Pulse Intervals (IPIs) for a Pre-Pulse to suppress jump response to a subsequent Pulse stimulus in a combined PPI stimulus we tested the effect of a range of biologically relevant IPIs (IPI: 5–1000 ms) on jump response frequency [56, 57]. The results clearly demonstrated that adult *Drosophila* – regardless of eye colour – exhibit Pre-Pulse Inhibition in the range of 5–200 ms IPIs (Fig. 1f; Supplementary Fig. 10; Supplementary Table 2). The lowest jump frequencies to PPI stimuli were observed at IPIs of 5–50 ms, indicating that the strongest PPI occurred in this range. Jump frequency gradually increased with longer IPIs, reaching the same level as the Pulse-alone response at an IPI of 200 ms. This indicates that PPI performance gradually declined from 5–200 ms (Fig. 1f, g). At even longer IPIs (400–1000 ms), neither PPI nor Pre-Pulse Facilitation was observed (Supplementary Fig. 8).

The data also showed that although flies with different eye colours require different initial light intensities and may exhibit varying jump frequencies in response to Pulse stimuli (Fig. 1f), they display similar PPI characteristics (Fig. 1f, g; Supplementary Fig. 8,9).

Here, we report for the first time that adult *Drosophila* exhibit light-off jump response PPI with properties comparable to those observed in higher organisms. Eye colour is an important variable: the closer the eye colour is to wild-type red, the higher the Initial Light Intensity required to elicit a sufficient number of jumps for PPI measurement. Brownish-red and red-eyed flies exhibited low jump frequencies to their optimized Pulse stimuli, which limited our ability to analyse the effects of different IPIs; nonetheless, these flies still displayed strong PPI (Supplementary Fig. 10; Supplementary Table 2).

To avoid adjusting light settings for different eye colours, we introduced 1 or 2 copies of the *GMR-wIR* element into the fly genomes, rendering the eyes weak-yellow or white (see Supplementary Table 1).

### Analysis of light-off jump response PPI data

The canonical measure of PPI is the PPI Index [58], calculated as a percentage reduction of the response to the Pulse stimulus:

$$\,{\boldsymbol{PPI\; Index}}( \% )={\bf{100}}-\left(\frac{{\boldsymbol{P}}{\boldsymbol{P}}{\boldsymbol{I}}{\boldsymbol{s}}{\boldsymbol{c}}{\boldsymbol{o}}{\boldsymbol{r}}{\boldsymbol{e}}}{{\boldsymbol{P}}{\boldsymbol{u}}{\boldsymbol{l}}{\boldsymbol{s}}{\boldsymbol{e}}{\boldsymbol{s}}{\boldsymbol{c}}{\boldsymbol{o}}{\boldsymbol{r}}{\boldsymbol{e}}}\times {\bf{100}}\right)$$

PPI Index (%) = 100% = Complete Pre-Pulse Inhibition

PPI Index (%) = 0% = No Pre-Pulse Inhibition

The PPI scores expressed as Relative Jump Frequency (referred to as Jump Frequency, too) in Fig. 1f are also presented in PPI Indices in Fig. 1g and Supplementary Fig. 11b.

In the literature PPI result calculations are based on the assumption that the startle response has an approximately Gaussian distribution [57, 59–62]. This assumption was found to be false in rats where the startle response distribution is better represented by a log-normal distribution [63]. However, our startle data showed binomial distribution (0 or 1 jump), therefore we employ a logistic regression model to analyse the effect of the preceding Pre-Pulse on the jump response to Pulse.

### Logistic regression analysis of PPI

**Fig. 2:**
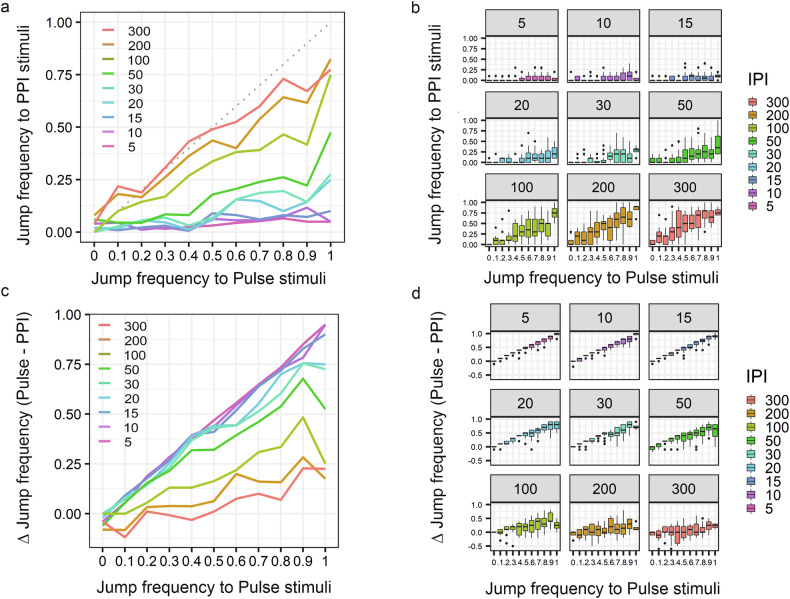
Dependency of the Jump frequency to PPI stimuli on the Jump frequency to Pulse stimuli. **a** Line plot showing the mean Jump frequency in response to PPI stimuli as a function of Jump frequency in response to Pulse stimuli at different Inter Pulse Intervals (IPIs). Lower values indicate a stronger PPI effect. **b** Boxplot showing the distributions of jump frequency measurements corresponding to the mean frequencies shown in **a**. **c** Line plot of the difference between Jump frequency in response to Pulse stimuli and Jump frequency in response to PPI stimuli (i.e., reduction in jump frequency due to PPI) as a function of Jump frequency to Pulse stimuli at different IPIs. Higher values indicate a stronger PPI effect. **d** Boxplot representation of the results shown in **c**. Data were collected over two years using white-eyed control flies (n = 1512).

In our experiments, groups of flies typically include individuals with a Relative Jump Frequency ranging from 1 (10 jumps in 10 Pulse trials) to 0 (0 jumps in 10 Pulse trials). Flies with a low Relative Jump Frequency in response to Pulse stimuli may not clearly exhibit Pre-Pulse Inhibition (PPI), or do so only at low resolution, which can reduce the statistical power for comparing different groups. We therefore investigated whether this variability influences the PPI jump response and subsequent analysis. We sub-grouped a set of control flies into categories based on their jump frequency to Pulse stimuli and plotted their jump frequency to PPI stimuli in all tested IPIs. We observed that flies with low jump response to Pulse stimuli do not show as efficient suppression of jump response to PPI stimuli as flies with higher jump response to Pulse stimuli (Fig. 2a, b). This effect is better visualized by plotting the jump frequency data as Pulse *vs* Pulse-minus-PPI (Fig. 2c, d). The higher the difference, the better the suppression.

From these comparisons, we concluded that the reduction in jump response to PPI stimuli — i.e., the manifestation of PPI — is confounded by the fly’s baseline jump response to Pulse stimuli. Therefore, this factor should be controlled for in the analysis. Such a statistical approach would allow us to include the “low jumpers” in the analysis, as they also carry valuable information. Although their PPI response is of lower resolution, they still exhibit PPI.

Because of the binary character of the jump response data, we applied a logistic regression model using a generalized linear mixed-effects model (GLMM) framework [64] in order to assess the effect of a variable of interest, e.g. genotype, on jump response following Pre-Pulse, Pulse or their combination. This framework allowed us to control for any bias caused by the experimental design, such as, individual flies, testing day, and behaviour system, which were included in the model as random effect variables. In order to control for the Pulse-PPI correlation, we also included in the model estimating the response to PPI stimuli an interaction term between response to Pulse and PPI stimuli at different IPIs. Details of the two statistical models — one for responses to Pulse or Pre-Pulse stimuli alone, and another for PPI — are provided in the Supplementary Materials (see “Logistic Regression Analysis of PPI”).

**Fig. 3:**
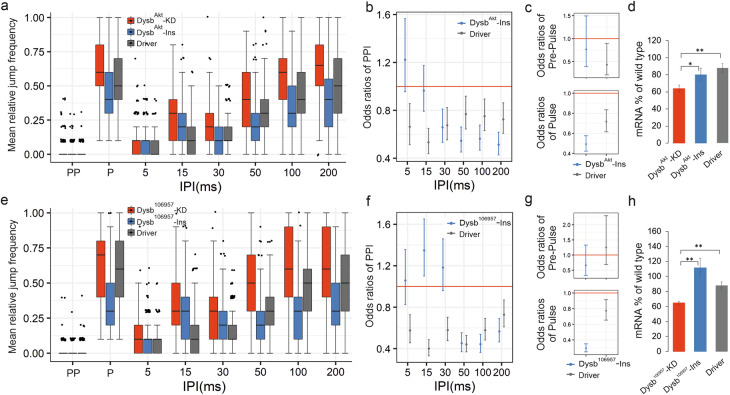
Knock-down mutants of schizophrenia susceptibility gene orthologue *Dysbindin* show PPI phenotype. **a** and **e** Jump frequency of *Dysbindin* RNAi Knock-down flies (*Dysb*^*Akt*^-KD, n = 242 and *Dysb*^106957^-KD, n = 248, respectively) compared to the appropriate control genotypes, *UAS-Dysb-RNAi* inserts alone (*Dysb*^*Akt*^-Ins, n = 235 and *Dysb*^*106957*^-Ins, n = 230, respectively), as well as *2xGMR-wIR*; *elav-Gal4, UAS-Dicer-2* (Driver, n = 248 and n = 253, respectively) alone in PPI experiments. For detailed genotypes see Supplementary Table 1. (**b, c** and **f, g**) Control/mutant jumping odds ratios (genotypes as in **a** and **e**) following PPI, Pre-Pulse as well as Pulse stimuli estimated by GLMM. At odds ratio 1 the red horizontal line indicates equal odds of jumping for a mutant (*Dysb*^*Akt*^-KD or *Dysb*^106957^-KD) and a given control genotype. Odds ratios for the two control genotypes are calculated as control/mutant jumping odds separately. Therefore, for a given control genotype odds ratios below 1 indicate lower chance of jumping, and in the case of PPI stimuli, stronger PPI compared to the mutant (i.e., the mutant PPI is weaker). When the error bars representing the 95% confidence intervals do not overlap with the odds ratio = 1 red horizontal line, it is considered to be a sign of statistically significant difference at the 0.05 significance level. (**d** and **h**) Relative levels of *Dysb* mRNA, as determined by qPCR, in indicated genotypes. *: P < 0.05, **: P < 0.01.

**Fig. 4:**
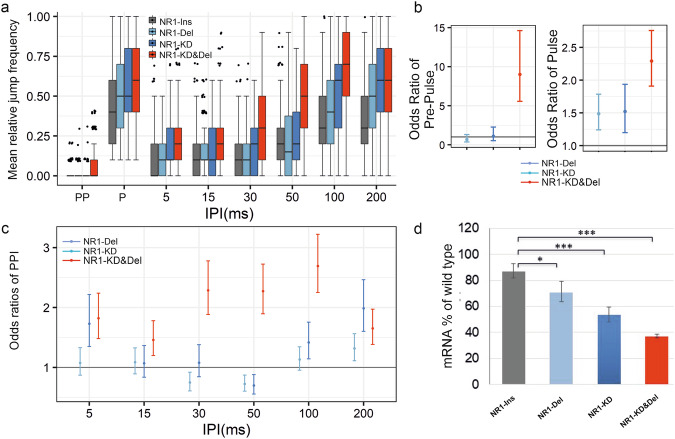
Down-regulation of *Nmdar1* (NR1) resulted in PPI phenotype. **a** Jump frequency of classical deletion (NR1-Del, n = 258), RNAi Knock-down (NR1^41666^-KD, n = 125) and combined NR1-KD&Del (n = 253) mutants of *Nmdar1* compared to control, UAS-NR1^41666^ insertion alone, (NR1-Ins) in PPI experiments. For detailed genotypes see Supplementary Table 1. **b** Pre-Pulse and Pulse performances of *Nmdar1* mutants expressed in odds ratios estimated by GLMM. Odds ratios for each mutant genotype are calculated as mutant/control jumping odds separately. At odds ratio 1 the grey line represents equal jumping odds for a given mutant and the common control, >1 odds ratio indicates higher odds of jumping for the mutant compared to the control. 95% confidence intervals are shown. **c** PPI phenotypes of *Nmdar1* mutants expressed in odds ratios. At odds ratio 1 the grey line represents the given IPI having the same effect on the chance of jumping for control and mutant, >1 odds ratio meaning higher odds of jumping, thus weaker PPI for the mutant genotypes. **d** Relative levels of *Nmdar1* mRNA in the different genotypes. *: P < 0.05, **: P < 0.01, ***: P < 0.001.

**Fig. 5:**
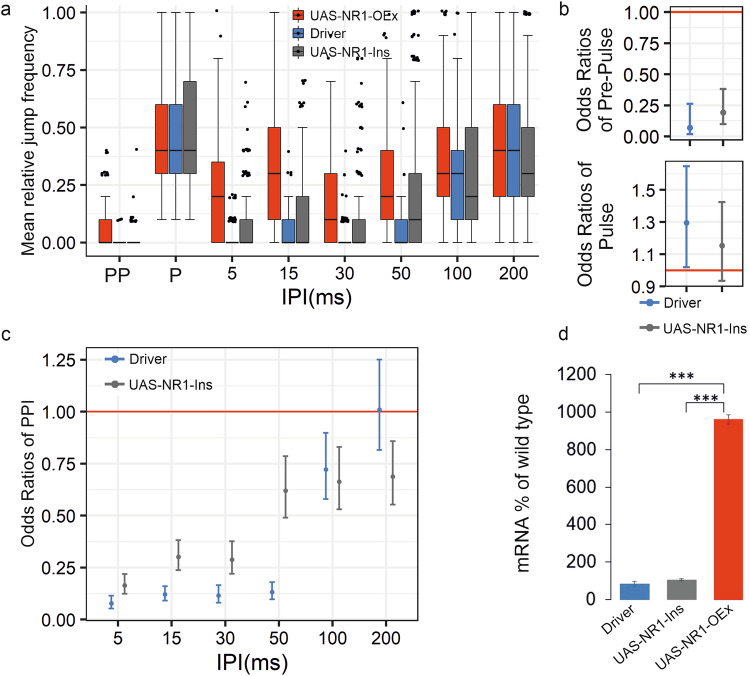
Up-regulation of *Nmdar1* (NR1) resulted in impaired PPI. **a** Jump frequency of full length NR1-cDNA neural overexpression transformant (UAS-NR1-OEx, n = 175) compared to two control genotypes (UAS-NR1-Ins, n = 177 and Driver, n = 161) at different IPIs. **b** Pre-Pulse and Pulse performances presented in odds ratios. **c** PPI performances presented in odds ratios. Control/mutant jumping odds ratios following PPI, Pre-Pulse as well as Pulse stimuli estimated by GLMM. At odds ratio 1 the red horizontal line indicates equal odds of jumping for the mutant (UAS-NR1_OEx) and a given control genotype. Odds ratios for the two control genotypes are calculated as control/mutant jumping odds separately. Therefore, for a given control genotype odds ratios below 1 indicate lower chance of jumping, and in the case of PPI stimuli, stronger PPI compared to the mutant. The error bars represent the 95% confidence intervals. **d** Relative levels of *Nmdar1* mRNA in the different genotypes. *: P < 0.05, **: P < 0.01, ***: P < 0.001. For detailed genotypes see Supplementary Table 1.

Jump response differences between two genotypes are estimated as odds ratios (e.g., see Fig. 3b, f). For a given genotype the odds of jumping is the frequency of jumping divided by the frequency of not jumping as a response to a given stimulus. The odds ratio represents the ratio of the odds values between two genotypes. In each comparison, the genotype represented by a single value is placed in the denominator. Accordingly, in Figs. 3 and 5, the ratios are calculated as control/mutant when a single mutant genotype is compared to two control genotypes, and as mutant/control in Fig. 4, where three mutant genotypes are compared to a single control genotype. With this model we found that n = 128 sample size per group (new 32 flies tested on 4 different days) is sufficient for reproducible results (Supplementary Fig. 11).

### Testing mutants in schizophrenia susceptibility genes for PPI defects

In neuropsychiatry, abnormal PPI is a widely accepted biomarker in humans and mammalian models as PPI measurements can reveal disorder states [4]. Hence, we asked whether perturbing *Drosophila* orthologues of human schizophrenia susceptibility genes would affect light-off jump response PPI. For this, we used the UAS-Gal4 binary system [65] in combination with the panneuronally expressed *elav-Gal4* driver to knock-down *Dysbindin* [35] and *Nmdar1* [28] by inducible RNAi. Our Gal4 driver line (Driver) also carried two additional genetic elements: 2x*GMR-wIR* to render the flies white-eyed [53, 54] and *UAS-Dicer-2* to enhance the effectiveness of RNAi [66]. Consequently, experimental flies contained *elav-Gal4*, *UAS—*gene-of-interest*—RNAi*, 2x*GMR-wIR* & *UAS-Dicer-2* (for full description of genotypes see Supplementary Table 1). These flies were compared to “*UAS—*gene-of-interest*—RNAi* insert alone” and “Driver alone” Controls. We confirmed RNA silencing by measuring mRNA levels of the targeted genes by qPCR.

### Knockdown of *Dysbindin* causes a PPI defect in *Drosophila*

Knocking down *Drosophila Dysbindin* gene with RNAi construct *Dysb*^*Akt*^ showed significantly reduced PPI at IPI range of 30–200 ms (Fig. 3a, b). Knock-down with a second, independent and overlapping, RNAi construct *Dysb*^*106957*^ (for genotype see Supplementary Table 1) generated consistent results of reduced PPI at IPIs 50–200 ms (Fig. 3e, f). Furthermore, we observed increased jump frequency to the Pulse stimuli in both cases of *Dysb*^*Akt*^-KD and *Dysb*^*106957*^-KD (Fig. 3c, g lower panels). For GLMM-calculated p values of statistical significance see Supplementary Table 3. The reduced PPI correlated well with the reduced mRNA levels of *Dysb* in the knock-down flies (about 60% of wild-type with both RNAi constructs; Fig. 3d, h). For RNA levels, p values of statistical significance see Supplementary Table 4. In conclusion, the downregulation of *Dysbindin* is characterised by phenotypes including PPI defects and “hyper-reactivity” to Pulse stimuli.

### Knockdown of *Nmdar1* causes a PPI defect in *Drosophila*

Considering the glutamate hypothesis of schizophrenia, we analysed the effect of modulating *Nmdar1* transcript levels on PPI in *Drosophila* (Supplementary Fig. 12). To knock down the *Drosophila Nmdar1* gene two *UAS-RNAi* constructs were used. With respect to *Nmdar1*^*37333*^-KD we observed reduced PPI phenotype in the range of 50–200 ms IPIs. Meanwhile, in *Nmdar1*^*41666*^-KD flies reduced PPI phenotype was observed at 5, 100 and 200 ms IPIs (Supplementary Fig. 12b,f; p values in Supplementary Table 3). This relatively weak PPI phenotype was obtained at *Nmdar1* mRNA levels of 49 and 58%, respectively (Supplementary Fig. 12d,h; p values in Supplementary Table 4).

Nevertheless, we wanted to generate a *Nmdar1* disease model with stronger PPI phenotype. This could be achieved by introducing a heterozygous *Nmdar1* deletion to further deplete the mRNA level in *Nmdar1*-KD flies. For this purpose, we chose to work with the 21-mer sort hairpin RNA expressing *Nmdar1* RNAi construct (*UAS-Nmdar1*^*41666*^_,_ instead of the long RNAi sequence-containing *UAS-Nmdar1*^*37333*^ one) to avoid the necessity of using *UAS-Dicer-2* in flies that will harbour multiple genetic elements. Hence, we introduced a heterozygous *Nmdar1* deletion into the *Nmdar1*^*41666*^-KD background (for full genotypes see Supplementary Table 1).

Figure 4 shows that *Nmdar1*-Deletion heterozygote flies (NR-Del), similarly to *Nmdar1*^*41666*^-KD (Nmdar1-KD) flies show weak PPI phenotypes, while the *Nmdar1*-KD&Del genetic combination results in a strongly reduced PPI phenotype at all IPIs (Fig. 4a, c). This is in accordance with the determined *Nmdar1* mRNA levels, where the lowest mRNA level (below 40%) was measured for the *Nmdar1*-Del&KD combination (Fig. 4d). We conclude that strong *Nmdar1* down-regulation causes severe PPI defect. Similarly to *Dysbindin* knock-down, an increased jump response to Pulse stimuli was observed in all the *Nmdar1*-KD, *Nmdar1*-Del and *Nmdar1*-KD&Del flies (Fig. 4b, right panel). In addition, *Nmdar1*-KD&Del flies respond more to Pre-Pulse stimuli than the control ones (Fig. 4b, left panel).

### *Nmdar1* overexpression causes a PPI defect in *Drosophila*

We wanted to address the question if there is an optimal range of *Nmdar1* mRNA level, required for PPI, by also overexpressing it in *Drosophila* [29, 67]. We expressed full-length *Nmdar1*-cDNA from a UAS construct driven by *elav-Gal4*. In these transformant flies the expression level of *Nmdar1* mRNA was about 10-times higher than in wild-type flies (Fig. 5d).

The flies with this very high level of Nmdar1 expression showed impaired Pre-Pulse Inhibition at 5–100 ms IPIs (Fig. 5a, c). In this case higher jump frequency is observed for the Pre-Pulse stimuli, only (Fig. 5b, upper panel). In conclusion, similarly to knock-down, up-regulation of *Nmdar1* resulted in impaired PPI, although in this latter case flies showed mild hyper-reactivity towards the Pre-Pulse stimulus.

## Discussion

Here we report a semi-automated, high-throughput light-off jump response behaviour paradigm for measuring Pre-Pulse Inhibition in adult *Drosophila*. One operator is able to simultaneously manage up to 32 single-fly tests in a 30 min period. There are great advantages of this relatively high-throughput adult PPI measurement method in genetic or compound screens over the labour intensive, expensive and ethically questioned vertebrate (rodent and zebrafish) model-based PPI methods. It is a general problem in measuring behaviour that the results are hard to compare due to the different and unique paradigms used to obtain them [21, 63]. To enable the research community to measure *Drosophila* habituation and PPI in the same platform, Aktogen Limited, Cambridge, UK made this Light-off jump response system commercially available.

It was demonstrated that modelling neuropsychiatric disorders (NPDs) in flies can have both mechanistic and predictive validity [19]. As Pre-Pulse Inhibition is a widely used endophenotype of these disorders, our highly efficient *Drosophila* PPI paradigm can support applying the potentials of this genetically tractable model organism to better understand NPDs and develop effective treatments for them [21].

In our behaviour system we demonstrate for the first time that the light-off jump response Pre-Pulse Inhibition (PPI) phenomenon exists in adult *Drosophila* and exhibits similar properties to those of higher organisms. PPI at Inter Pulse Intervals (IPI, also known as Lead Interval) in the range of 5–200 ms were observed, overlapping with the range of 30–500 ms in humans [56] and the typical range of 2–500 ms in rodents [57]. These parallels in IPIs further suggest the usefulness of the adult *Drosophila* PPI system in reducing experiments on mammalian models.

The amplitude of the Pre-Pulse stimulus also plays a significant role in PPI. Higher-intensity Pre-Pulses generally lead to greater inhibition of the startle response. This relationship was observed in studies where increased Pre-Pulse intensity correlated with enhanced PPI, suggesting a linear relationship between Pre-Pulse intensity and the magnitude of PPI [68, 69]. In this study – aimed at demonstrating the presence of PPI in adult *Drosophila* – we standardised the Pre-Pulse amplitude across all strains at the lowest level that elicited a minimal response. In follow-up studies, we will further optimize PPI parameters to identify specific “sweet spots” for each genotype.

The high-throughput nature of our PPI method allows to test PPI at multiple IPIs in one experiment. This feature will allow more detailed descriptions of the effects of genetic mutations and/or chemical compounds on the underlying cognitive processes, with the potential to identify distinct molecular and/or neural mechanisms of these processes.

In *Drosophila* larval PPI, where there was no measurement below 100 ms IPI, the best PPI was detected at 300 ms IPI [23]. Meanwhile, in adults we could not measure PPI over 200 ms IPI. We speculate that the difference in the PPI characteristics in the two developmental stages originates from the way in which the nervous system processes information. Moreover, it may also matter that the startling/PPI stimuli travel differently in the two behaviour systems (light for the adult *vs* sound – possibly in the agar base – in case of the larval assay).

A paper on PPI in adult *Drosophila* was recently published by Schiöth et al. However, the testing parameters in that study differ markedly from those generally accepted as standard PPI experimental conditions [70]. Although PPI is typically measured by modulating the startle response, it is notable that their light-off stimulus elicited a movement response with a reaction time approximately 100 times slower — and a speed about 1000 times slower — than the well-characterized *Drosophila* jump-and-flight startle response [25, 50, 71]. Additionally, the durations of the effective IPIs in their study were approximately 10–1000 times longer than those typically used in mammalian PPI studies [57, 72–74] as well as in other PPI studies [61, 75–77]. Upon closer examination of their results, we did not find the evidence for a genuine PPI phenomenon to be convincing.

While analysing light-off jump response PPI data we noticed that the jump response of each individual fly to PPI stimuli was dependent on its jump response to Pulse stimuli, as a Pulse response is prerequisite for the Pre-Pulse to show suppression. This confounded a proper assessment of PPI performance by the conventional PPI index, which is in accordance with earlier findings in human and mouse experiments [78] and recently published results obtained in rats [63]. To circumvent this problem we applied a logistic regression model using a generalized linear mixed-effects model (GLMM) framework [64] on the PPI data. This model was designed to control for potential confounding factors on PPI such as i) jump response to Pulse alone, ii) batch effects caused by the experimental design, iii) individual differences between flies. We propose to apply this approach to the analysis of *Drosophila* PPI data with a binomial distribution (0 or 1 jump in this case). Furthermore, we propose that the same approach might be valuable in the analysis of PPI data with different distributions, where there are also needs to correct for effects of experimental design and individual differences. The R-based PPI data analysis software will be made available to researchers interested in analysing PPI data with the proposed method (see “Code availability”).

We show that our light-off behaviour system can monitor changes in PPI performances in genetically manipulated *Drosophila*. We were able to detect PPI phenotypes by decreasing or increasing the expression of two *Drosophila* orthologues of human schizophrenia susceptibility genes (*Dysbindin* [32–35] and *Nmdar1* [27, 28, 30, 31]). Panneural down-regulation of *Dysbindin* and *Nmdar1* transcription resulted in a reduced PPI phenotype, which is in good accordance with results obtained in rodent schizophrenia models and humans [36–39, 47, 48]. Interestingly, *Nmdar1* overexpression also resulted in a PPI deficit. This suggests that deviation from an optimal range of relevant gene activity in any directions can be decremental to cognitive processes, as it is proposed earlier [79].

When attempting to introduce *Drosophila* into the candidate gene and NPD drug discovery process, its role can be best placed as the first whole animal model after in vitro screens or replacing them, and before rodent model tests. Both target validation and lead optimisation steps could be performed in “humanized flies”, in which the human orthologue of the fly gene can be expressed *in lieu* of the original *Drosophila* gene [80]. We propose that it is readily possible to employ various approaches to help unravelling the genetic, molecular, cellular and system biology components of NPDs in fly models.

We hope that our work can contribute to finding solutions for the treatment of such debilitating mental disorders as Schizophrenia [81] and Bipolar Disorder [82]. It further may contribute to develop treatment for Neurodevelopmental disorders including Autistic Spectrum Disorder [83], Attention Deficit Hyperactivity Disorder (ADHD) [84], and monogenic conditions such as Fragile-X syndrome [85], all known to affect PPI performance in human patients.

The applications of the *Drosophila* light-off jump response PPI paradigm can include large-scale genetic screens and efforts to identify PPI-related novel molecular/genetic components, even testing multiple combinations of mutations in mental disorder related genes. An advantage of our assay is that the light-off jump circuitry is almost completely described [71, 86] and there are tools available to target the individual components, facilitating mechanistic studies and treatment target identification.

Oscillations in the alpha, beta, theta, and gamma bands are integral to the neural mechanisms underlying PPI. These oscillations enable dynamic gating of sensory and motor information, and their modulation is influenced by attention and clinical factors [87, 88]. Disruptions in these oscillatory patterns can impair PPI and are associated with various neuropsychiatric disorders [89].

Studies have shown that beta-range oscillations (20–30 Hz) in the *Drosophila* brain are associated with selective attention to salient visual features [90]. These oscillations are phase-locked to attended stimuli, suggesting a role in regulating attention and sensory salience [91]. The presence of beta oscillations during attention tasks in *Drosophila* suggests that oscillatory activity may be a conserved mechanism for filtering and prioritizing sensory information. This is similar to findings in mammals, where beta oscillations play a role in attention and sensory gating and may provide another method to study PPI mechanism and treatment approaches in *Drosophila*.

## Methods

### Automated light-off jump response habituation and Pre-Pulse Inhibition system

Light-off jump reflex habituation & PPI system flow chart:
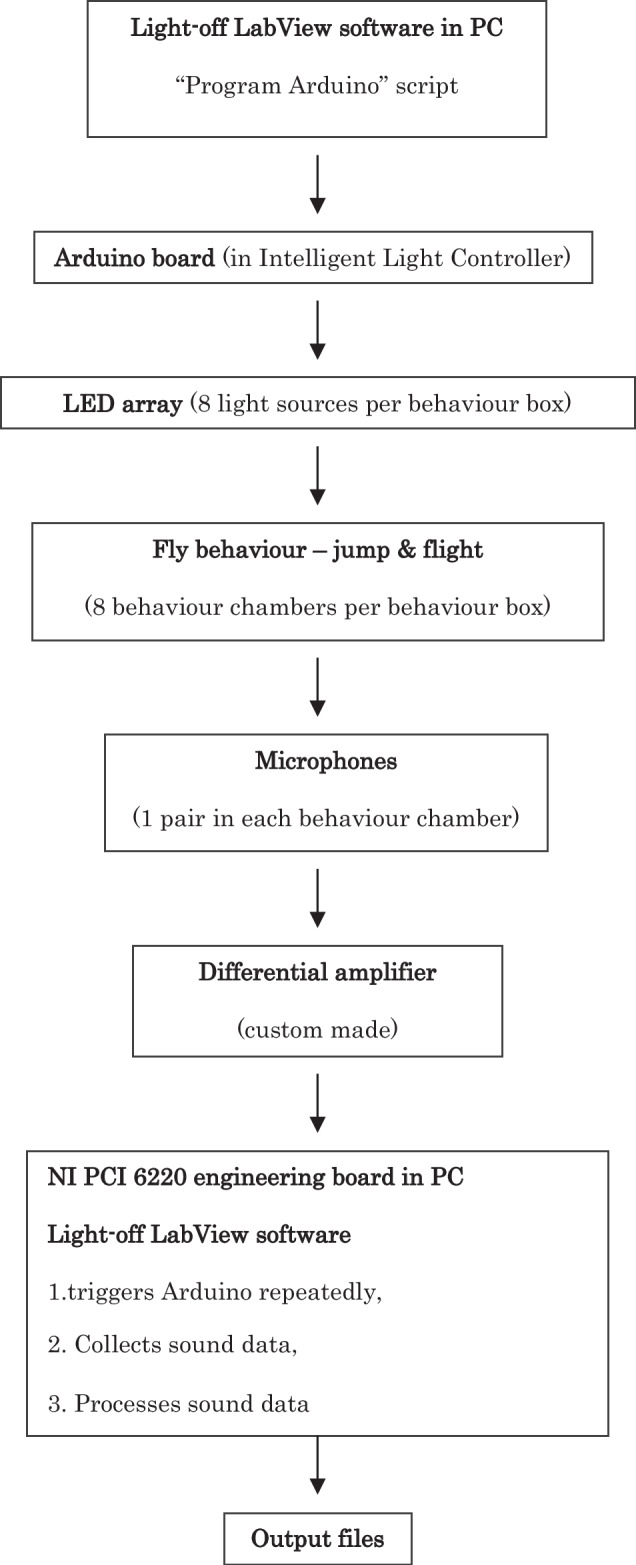


The output of the sound analysis indicates whether a jump occurred (“yes” or “no”) for the flies in each chamber. If the recorded sound volume exceeds the experimenter-defined threshold, the event is classified as a jump, and a value of ‘1’ is entered into the output data file for the corresponding time point/trial and chamber. If the volume does not exceed the threshold, a value of ‘0’ is recorded (see Supplementary Figure 2).

The jump threshold is set across all experiments safely above the observed noise level (sound signal without flies in the chambers).

Automated scoring of jump responses via audio recording was previously validated against manual scoring using simultaneous audio and video [26]. The results showed 93.5% agreement, with 4% of jumps detected only on video (“small jumps, below threshold”, false negatives) and 2.5% only by audio (higher noise incidences, above threshold, false positives). Both false positive and negative jumps can be reduced in a low-noise environment where a lower jump threshold can be set, and accidental noises do not occur.

Another type of false positive jump can occur when flies jump despite no change in light intensity. As shown in Fig. 1d, the probability of this happening is less than 1%. To ensure that jumps are closely associated with light-dimming stimuli, jumping activity is monitored only for 500 ms following the onset of a light-dimming stimulus.

The output files were analysed using a custom-made, R-based PPI analysis software, which implements a generalized linear mixed-effects model (GLMM).

### Fly stocks

Flies were maintained at 25 °C on standard cornmeal-yeast-agar medium at a 12 h light-dark cycle. Fly husbandries were performed using standard genetic techniques. Wild-type isogenic Canton-S *w*^*1118*^ (Iso31) [92] was from Steven Russel lab, University of Cambridge, UK, Department of Genetics. Three RNAi silencing constructs, 1. *Nmdar1* RNAi, stock #37333 (*w*^*1118*^**;** *P{GD2808}v37333*), 2. stock #37334 (*w*^*1118*^**;** *P{GD2808}v37334*) and 3. *Dysbindin* RNAi gene silencing construct, stock #106957 (*w*^*1118*^; *106957* *P{KK102468}VIE-260B*) were generated by Vienna Drosophila Resource Center (VDRC). The following stocks were obtained from Bloomington Drosophila Stock Center (BDSC): 1. *Nmdar1* RNAi gene silencing construct generated by Transgenic RNAi Project (TRiP) stock #41666 (y1 sc* v1; P{TRiP.HMS02199}attP2), 2. deletion including *Nmdar1* gene, stock#23146 (*w*^*1118*^*; Df(3* *R)BSC179/TM6B, Tb1*), 3. the *UAS-Nmdar1* overexpression line #8275 (*y*, *w*^*1118*^; *UAS-Nmdar1*) and 4. white RNAi gene silencing construct stock# 32067 (w^*+*^, *GMR-wIR* [51]). The *Dysbindin*^*dsRNA-*A^ RNAi gene silencing construct was generated in our laboratory (for details see below). To silence the expression of *Nmdar1* and *Dysbindin* in the central nervous system we employed the pan-neural driver line *w*^*-*^, *elav-Gal4*^*c155*^ (BDSC stock# 458) and the 3^rd^ chromosomal *elav-Gal4* insertion as part of the UAS/Gal4 system. The *“w*^-^, *elav-Gal4*^*c155*^, *GMR-wIR*” recombinant line was established in our laboratory by meiotic recombination of the *“w*^-^, *elav-Gal4*^*c155*”^ and the “w^+^, *GMR-wIR*” insertions on the X chromosome. The *“w*^-^; *elav-Gal4, UAS-Dicer2*” recombinant line was generated by recombining of the *“w*^-^; *elav-Gal4*” and the *“w*^-^; *UAS-Dicer2*” insertions on the 3^rd^ chromosome.

For detailed description of genotypes in experiments see Supplementary Table 1.

### Setting up a scale for eye colour shades of flies with different genotypes

To determine eye colour shades in flies of different genotypes, head images of 5-day-old flies were captured using a Leica DMC2900 digital microscope camera (in colour mode) mounted on a Leica M165 FC stereomicroscope, controlled with LAS X Multi Channel Acquisition software.

The original images were converted into RGB (red-green-blue) and HSB (hue-saturation-brightness) stacks using FIJI (version 1.54p) [93] with a macro script. After selecting the central area of the eyes, the statistics of the region of interest (ROI) were extracted from the respective image layers to obtain average values for R, G, B and H, S, B. The analysis code is provided in the Supplementary materials.

### PPI behaviour experiments

The PPI experiments were performed under standardized conditions at 25 °C and 70% relative humidity. To obtain experimental flies either from crosses or strains, 10–15 inseminated females were let to lay eggs for 24 h in a bottle, and the resulted – typically male – offspring were collected by cold anaesthesia as freshly hatched (age of < 24 h) adult flies. After reaching 5–7 days of age, the flies were placed inside the behaviour chambers and allowed to adapt for 5 min before the behaviour experiment was started.

Light-off or dimming stimuli evoked a startle jump response in flies. A light-off/dimming stimulus was defined in terms of its changing level of light intensity and duration. In PPI experiments the Initial Light Intensity was chosen to be 6.36 lux (arbitrary light setting 50; see Supplementary Fig. 5) and the following stimuli were presented to the flies (see also Fig. 1c):

- Pre-Pulse (PP, 50→35 dimming, 15 ms duration),
- Pulse (P, 50→10 dimming, 15 ms duration) and
- “Pre-Pulse & Pulse” combined [PPI, 50→35 & 50→10 dimming, 15 ms duration for both stimuli with different durations of Inter Pulse Intervals (IPI)].

One PPI experiment consisted of 10 repeats of PP, 10 repeats of P and typically 6 × 10 repeats of PPI stimuli per fly, randomly ordered. In PPI combinations the PP and P stimuli were separated by different Inter Pulse Intervals of 5, 15, 30, 50, 100 and 200 ms. Each stimulus presentation was separated by a 5 s Inter Trial Interval (ITI), which ensures the subsequent trials to be completely independent of one another (see Fig. 1e).

In a typical PPI experiment different genotypes were tested on the same day. Each genotype was represented by 32 flies per experimental day. Each fly was typically exposed to 80 trials per experiment per day (lasting for about 7 min). As a result, in a typical 4-day or 6-day experiment the total number of stimuli presented was 320 or 480, respectively.

### Data analysis

Jump was defined as electronically amplified sound from the behaviour chamber higher in voltage than the experimentally set jump threshold. Accordingly, the PPI system generated output files containing “1” and “0” values for jump events and non-jump events, respectively (Raw jump data). From these values, Relative Jump Frequencies for each individual fly and stimulus type (i.e., PP, P and PPI) were determined with the help of a custom-made PPI analysis software utilizing base R functions [94], and the canonical PPI Index [58] was calculated according to the following formula:

$$\mathrm{PPI}\,\mathrm{Index}=100-\frac{\mathrm{PPI}\,\mathrm{score}}{\mathrm{Pscore}}* 100$$

where “PPI score” and “P score” values correspond to Relative Jump Frequency to PPI stimuli and Relative Jump Frequency to Pulse stimuli, respectively.

PPI Index = 0 indicates no Pre-Pulse Inhibition, PPI Index = 100 indicates perfect Pre-Pulse Inhibition (PPI). For pairwise visual comparison of Pulse and PPI scores of all genotypes tested see Supplementary Fig. 13.

Alternatively, the PP, P and PPI Relative Jump Frequencies were directly used in mixed-effects model analysis.

### PPI statistical analysis

The results give the sample sizes for the flies tested, after exclusions. According to pre-established criteria, flies that did not jump at all in response to the 10 Pulse stimuli, and/or flies that jumped 40% or more to the Pre-Pulse stimuli were excluded from the statistical analysis. No randomization was used to determine how flies were allocated to experimental groups. In one experiment of typically 32 flies contained always the same genotype. Each experiment was repeated at least 3 times in different days with different set of flies. The investigators were blinded to the group allocation during the investigation and when assessing the outcome (coded genotypes).

The effect of genotype on Relative Jump Frequencies was estimated as odds-ratios by using generalized linear mixed-effects models (GLMM). We used the ‘glmer’ function from the ‘lme4’ R package [64], with a binomial distribution for the jump data and a logit link function. Full description of the models are provided in the Results section ‘Logistic regression analysis of PPI’.

Statistical test results are presented either as P values and depicted in bar charts with median and standard error of the mean (s.e.m.), or as odds ratios and 95% confidence intervals using GLMM statistical tests.

### Molecular cloning of *Dysbindin* RNAi construct

A 585 bp long region (3 L: 18,868,047… 18,868,631; FB2022 release = r6.46) of the first exon of *Dysbindin* gene was PCR amplified with forward (5ʹ cagaTCTGTCGTCCAGCAGGAGCAGTAG3ʹ) and reverse (5ʹ cgtcgaCGCTGTTTGTACTCCTCCATATCC3ʹ) primers carrying a BglII and a SalI site at their 5’ ends, respectively. The resulting PCR product was inserted into the PCR cloning vector Topo-TA (Thermo Fisher Scientific, #450641) and subsequently transferred as BglII, SalI double digested fragment into BglII, XhoI sites of pWizMod[vector (GenBank accession number AB186054) [95]. In an additional cloning step, the same insert was cut out from Topo-TA by KpnI, XbaI double digestion and inserted into the KpnI, XbaI sites of the intermediate plasmid, resulting in *pDysbindin*^*dsRNA-A*^ with the desired inverted repeat arrangement. The plasmid was microinjected into *w*^*1118*^*; Sb, Δ2–3/TM6* embryos, and P-transposase-based integrants were selected for their mini-w^+^ eye colour.

### RNA preparation and quantitative real-time PCR (qRT-PCR)

Total RNA from twenty *Drosophila* heads for each genetic combination was purified using the RNA isolation kit of Macherey-Nagel (Macherey-Nagel, Düren, Germany). All the preparation steps were carried out according to the manufacturer’s instructions. RNA samples were stored at –80 °C in the presence of 30 U RiboLock RNase Inhibitor (Thermo Fisher, Waltham, MA, USA) for further analysis. The quantity of isolated RNA samples was checked by NanoDrop 3.1.0 (Thermo Fisher, Waltham, MA, USA).

1 μg of total RNA was reverse transcribed using the High-Capacity cDNA Archive Kit (Thermo Fisher Scientific, Waltham, MA, USA) according to the manufacturer’s instructions in a T100 Thermal Cycler machine (BioRad, Hercules, CA, USA). Briefly, the reaction mixture was the following: 10 μl (1 μg) total RNA template, 2 μl 10x RT Buffer, 0.8 μl dNTP, 2 μl Random primers and 1 μl Reverse transcriptase in 20 μl final volume. The temperature profile of the reverse transcription was the following: 10 min. at room temperature, 2 h at 37 °C, 5 min. on ice and finally 10 min. at 75 °C for enzyme inactivation.

After two times dilution, 1 μl of the diluted reaction mix was used as template in the qRT- PCR. The reaction was performed on a RotorGene 3000 instrument (Qiagen, Hilden, Germany) with gene-specific primers and qPCRBIO SyGreen Mix Lo-ROX mix (PCR Biosystems, London, UK) according to the manufacturer’s instructions at a final primer concentration of 250 nM under the following conditions: 2 min. at 95 °C, 40 cycles of 95 °C for 5 sec., 60 °C for 30 sec. Melting temperature analysis was done after each reaction to check the quality of the products. Primers were designed online using the Roche Universal Probe Library Assay Design Center or the Integrated DNA Technologies qPCR Assay Design RealTime PCR Tool. The quality of the primers was verified by MS analysis provided by Bioneer (Daejeon, South Korea). Individual threshold cycle (Ct) values were normalized to the mean Ct values of *DmRap, DmGiant, DmMnf* internal control genes. Relative gene expression levels are presented as percentage to wild type, calculated using the formula (fold change = 2^ΔΔCt^), according to the ΔΔCt method [96]. Information about the genes and the primers is summarized in Supplementary Table 5.

### Statistical analysis of qRT-PCR data

RNA samples were prepared and tested in four parallels (n = 4) for each genetic combination. Statistical comparison of normalized Ct (ΔCt) values of control and mutant genotypes was done by Student’s t-test (two-tailed, unequal variance). Results were summarized in Supplementary Table 4.

## Supplementary information

Pre-Pulse Inhibition of an escape response in adult fruit fly, Drosophila melanogaster

## Data Availability

The image converting macro code can be found in the Supplementary Material as “getColour_RGB_HSB_code”. The NI LabView-based Light-off Jump Response software (‘Light-off software’) and the R-based PPI data analysis software (a collection of custom-made R scripts constructed in-house) are components of the hardware-software package offered by Aktogen Ltd. The authors are willing to provide the PPI data analysis software along with the raw data upon request to facilitate processing.

## Data availability

Data are available from the authors upon reasonable request.
