# Supplementary material for: Pre-Pulse Inhibition of an escape response in adult fruit fly, Drosophila melanogaster
Source: Transl Psychiatry. 2026 Jan 8;16:22. doi: 10.1038/s41398-025-03717-5 (PMC12804964; doi:10.1038/s41398-025-03717-5)
Supplement: Supplementary file 1 — Pre-Pulse Inhibition of an escape response in adult fruit fly, Drosophila melanogaster [file 41398_2025_3717_MOESM1_ESM.docx]

**Supplementary Materials**


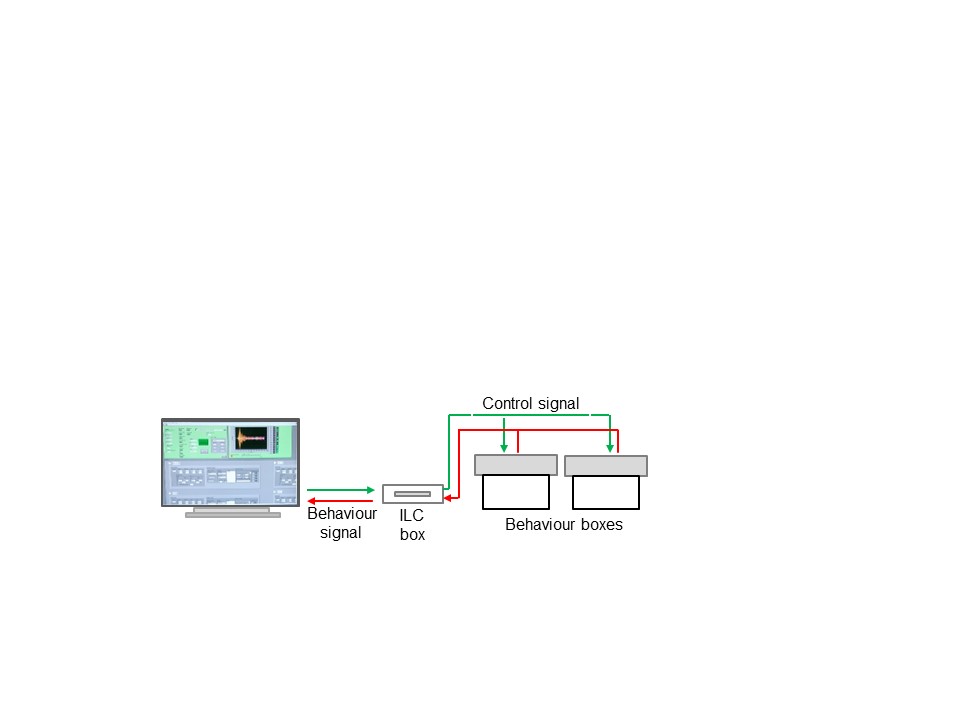
a

b


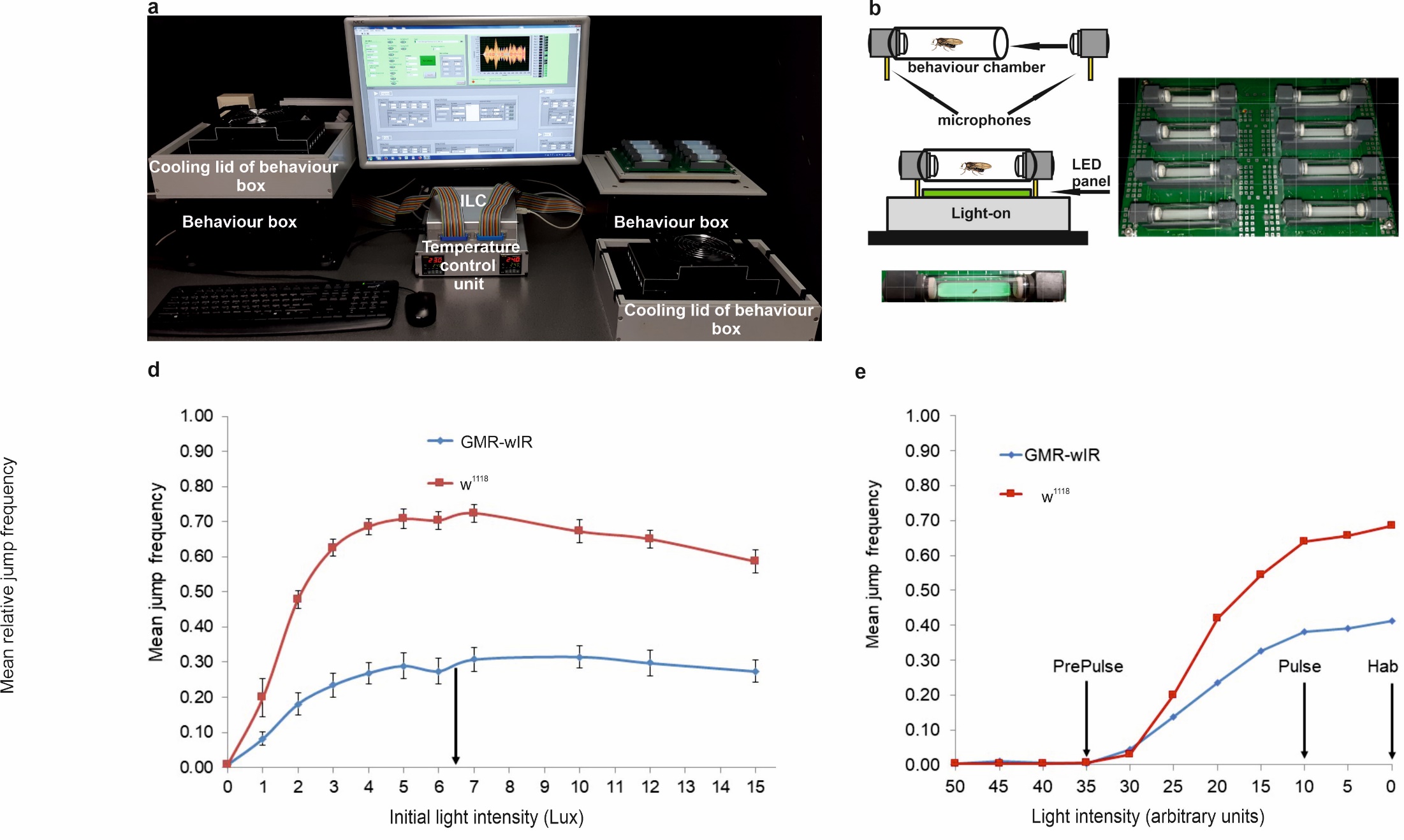


**
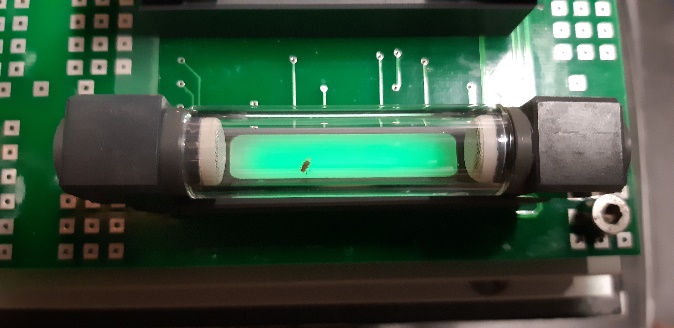
**c

**Supplementary Figure 1: Light-off jump reflex behaviour system.** **(a)** Schematic drawing of the experimental circuit. **(b)** One behaviour system with two behaviour boxes, Intelligent Light Controller (ILC), Temperature Control Unit and the user interface of the control software. A behaviour box consists of a Printed Circuit Board (PCB) with eight behaviour chambers each of positioned over a green light emitting diode (LED). **(c)** One behaviour chamber, flanked by microphones, used to test a single fly individually.

**Light-off jump reflex habituation & PPI system technical components**

**The system includes:**

- 2x Light stimulated behaviour boxes with 8 test-units. Each unit equipped with green light source, PTFE behaviour chamber, two microphones and amplifier.
- 1x Intelligent Light Controller Unit (ILC box) with Arduino light controller board
- 1x National Instrument PCI card 6220 in the PC;
- 2x Cable with a 6 pin Mate-N-Lok to 5 pin Lumberg industrial connector (to connect the Temperature Control Unit and the cooling lid of the behaviour box);
- 2x Ribbon cable (coloured) with a 39 pin D-sub connector on both sides (to connect the ILC box and the behaviour box);
- 1x SHC68-68-EPM Shielded Cable 68 D-Type to 68 VHDCI Offset (alternatively: SCSI2 VHDCI to HD68P M cable) (to connect the National Instrument PCI card 6220 with the ILC box)
- 1x “Light-off software” Nation Instrument LabVIEW control software.
- 1x R based Pre-Pulse Inhibition data analysis software
-
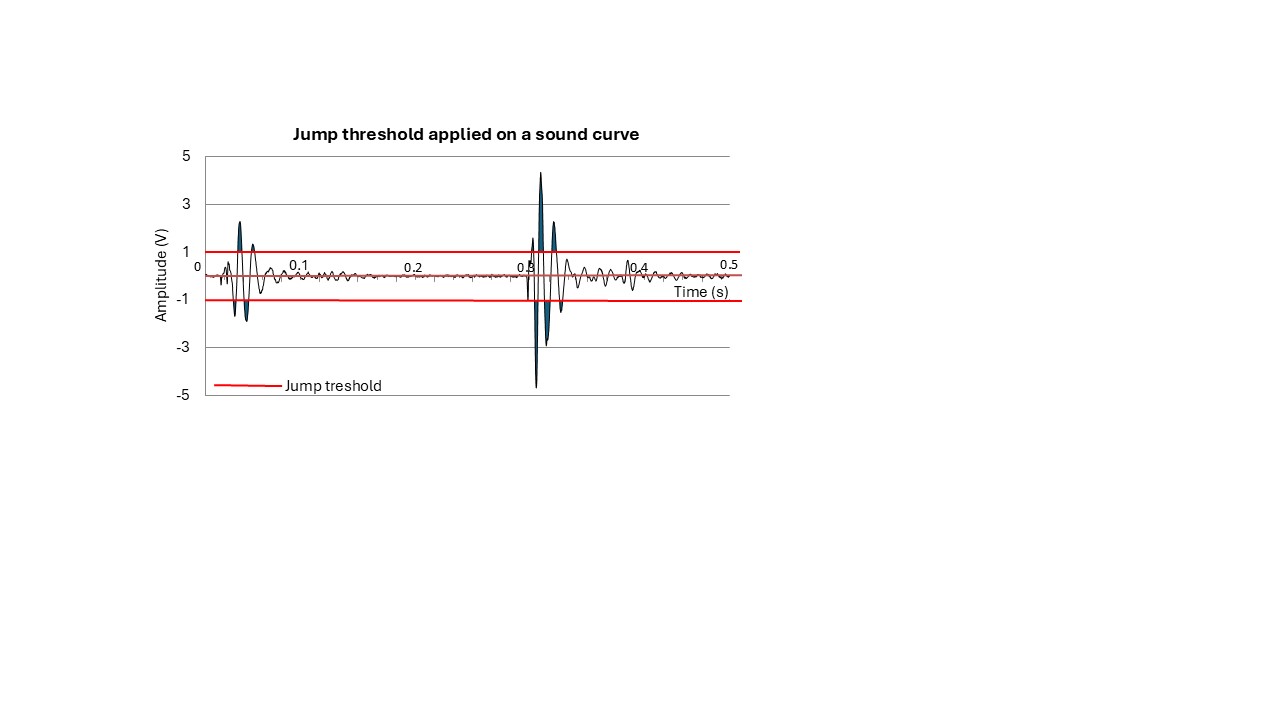


**Supplementary Figure 2**: **Empirically determined jump threshold applied to a sound trace over a representative 500 ms sample.** Sound levels exceeding the threshold are recorded as 1 (jump), regardless of the number of times the threshold is crossed.

**
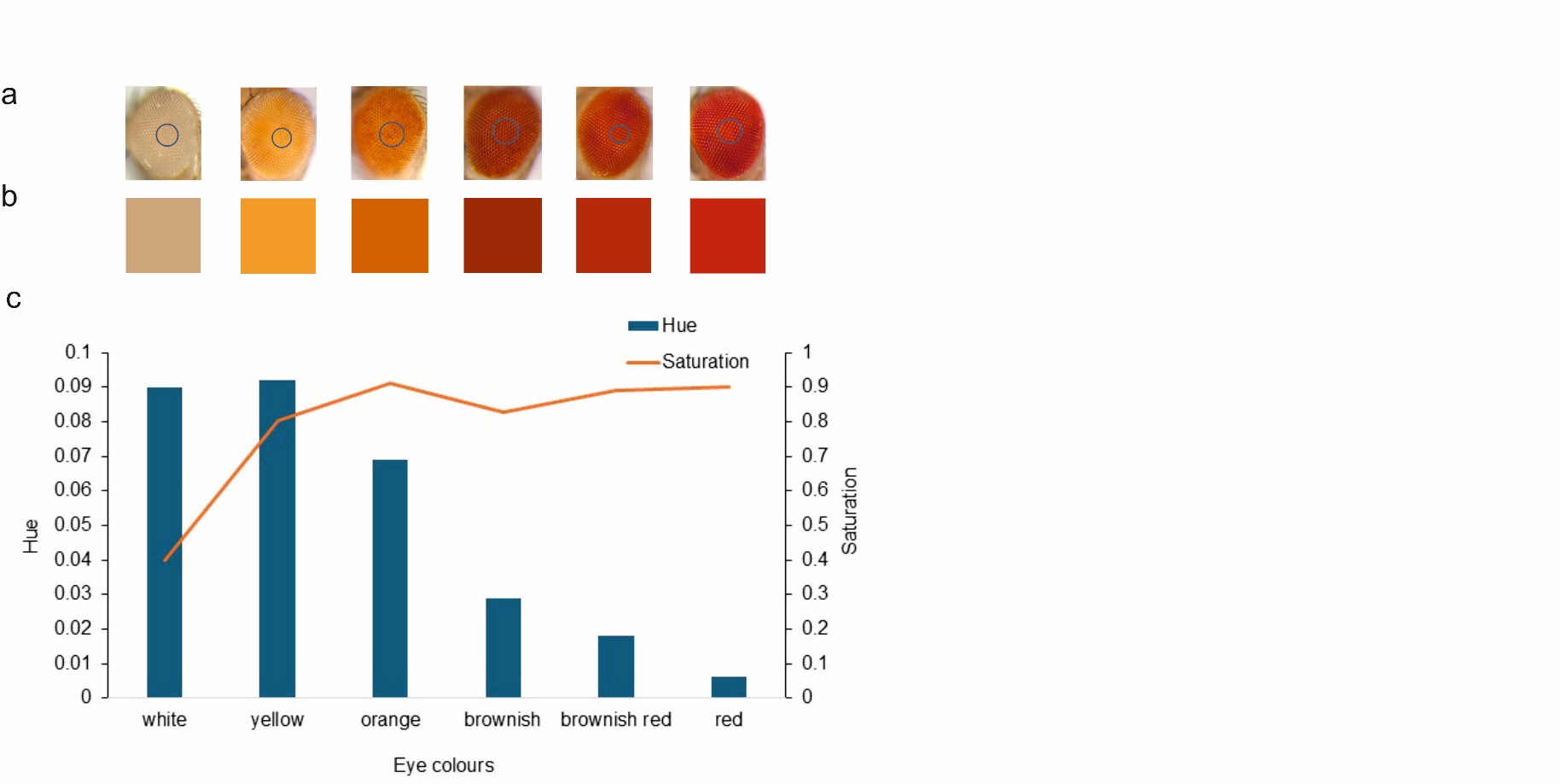
**

**Supplementary Figure 3: Eye colour scale.** **(a)** Images of Drosophila eyes with six different pigmentations. Blue circles indicate the regions used for calculating hue and saturation. **(b)** Colour squares were generated based on the average hue and saturation values calculated from the regions shown in (a). **(c)** Diagram displaying the average hue (bar chart) and saturation (line) values for the six eye colour categories. For calculation details see Methods.


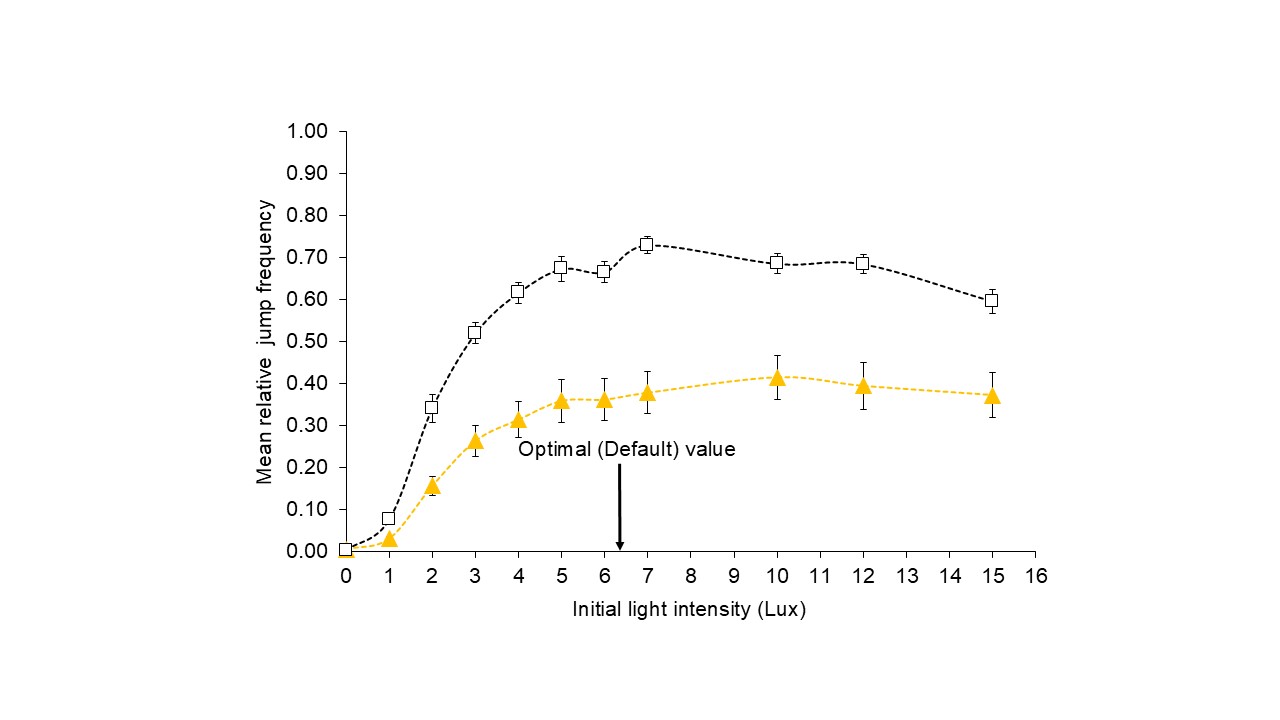


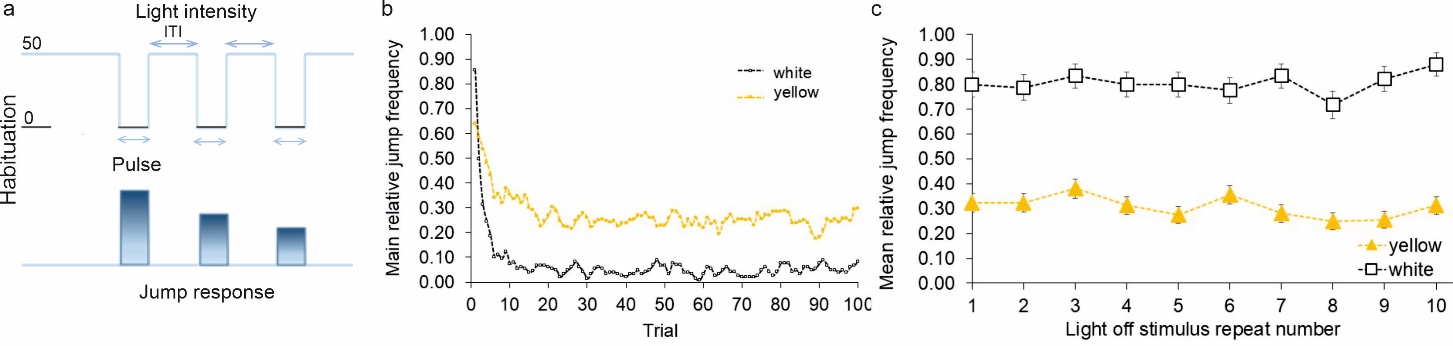


**Supplementary Figure 4: Jump response evoked by light-off stimuli in white-eyed and pale-yellow-eyed flies.** Initial light intensity range: 0 − 15 Lux; light-off stimulus: X🡪0 Lux. Mean values were calculated from ten repeats at each initial intensity. Black arrow points to 6.36 Lux, that was selected as the default light intensity for further light-off experiments.


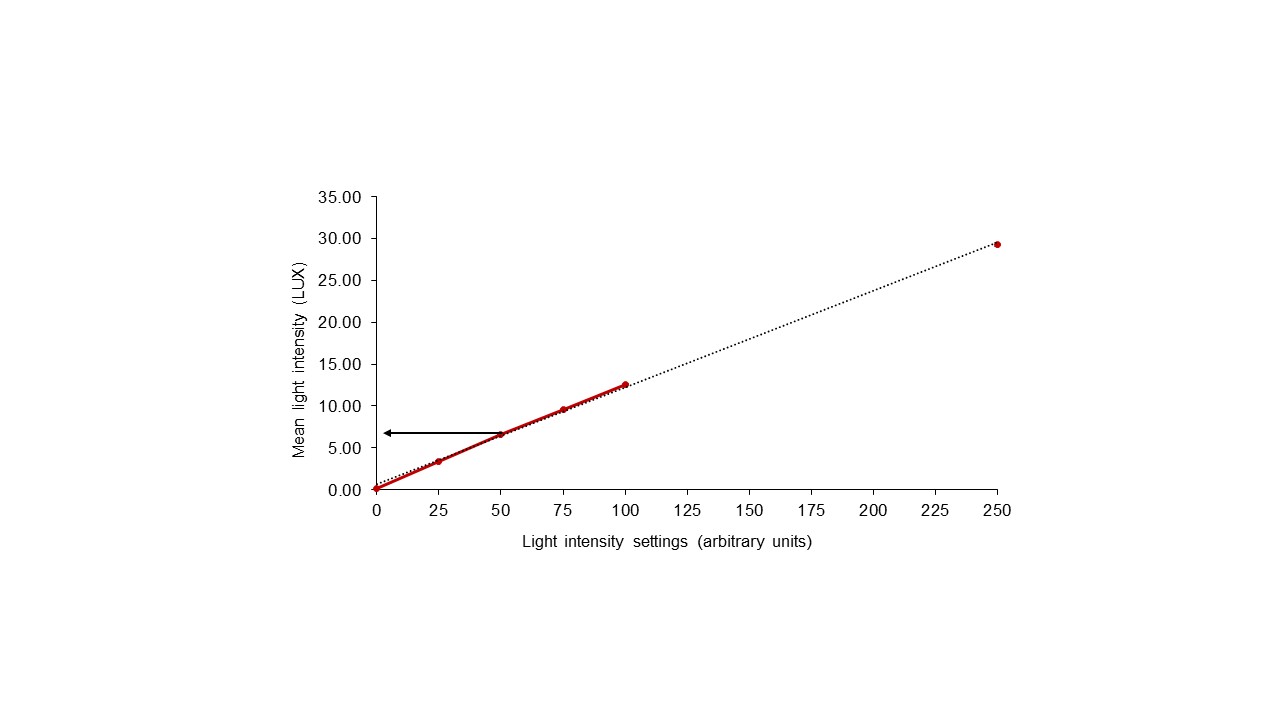


**Supplementary Figure 5: Measured light intensity vs. light intensity settings.** The measured light intensity values linearly correlate with the settings values. At 50 arbitrary unit black arrow points to the corresponding 6.36 Lux value, chosen from data represented on Supplementary Figure 4.


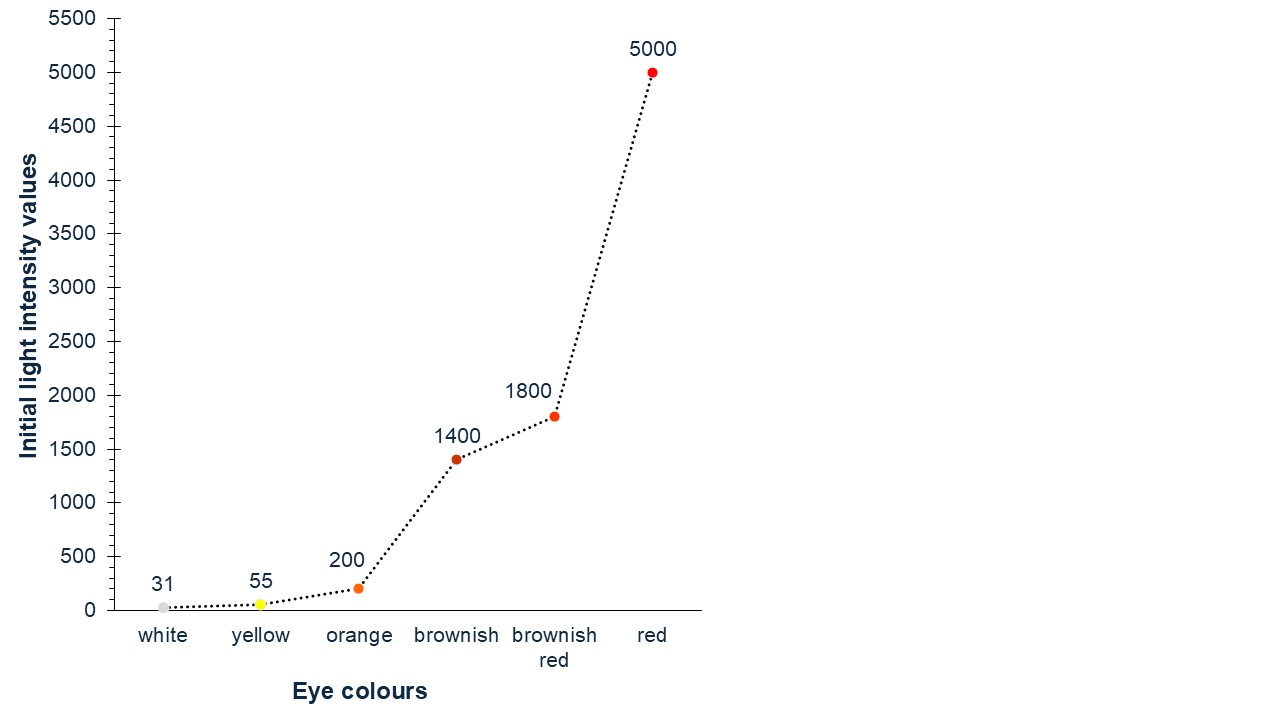


**Supplementary Figure 6: Correlation between initial light intensity values and eye colour.** Initial light intensity values (in arbitrary units, as defined in Supplementary Figure 5) at which fruit flies with different eye colours reach the plateau maximum of their Mean Relative Jump Frequency curves.


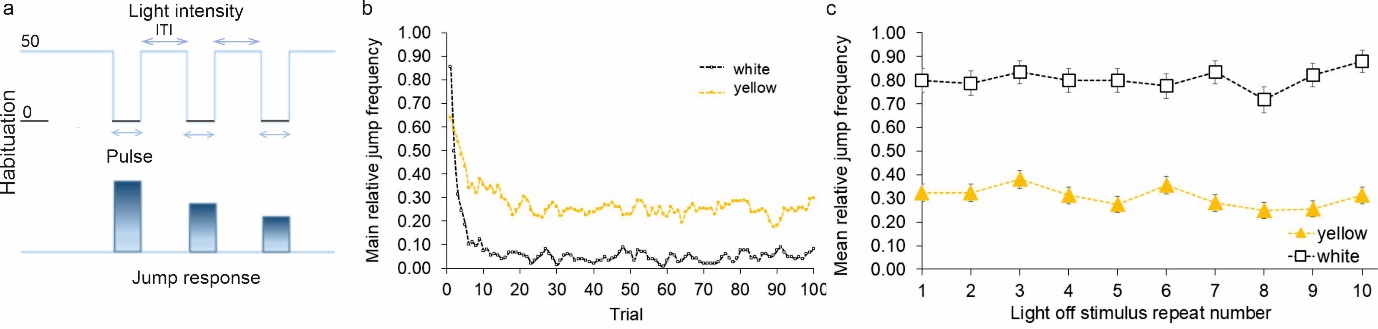


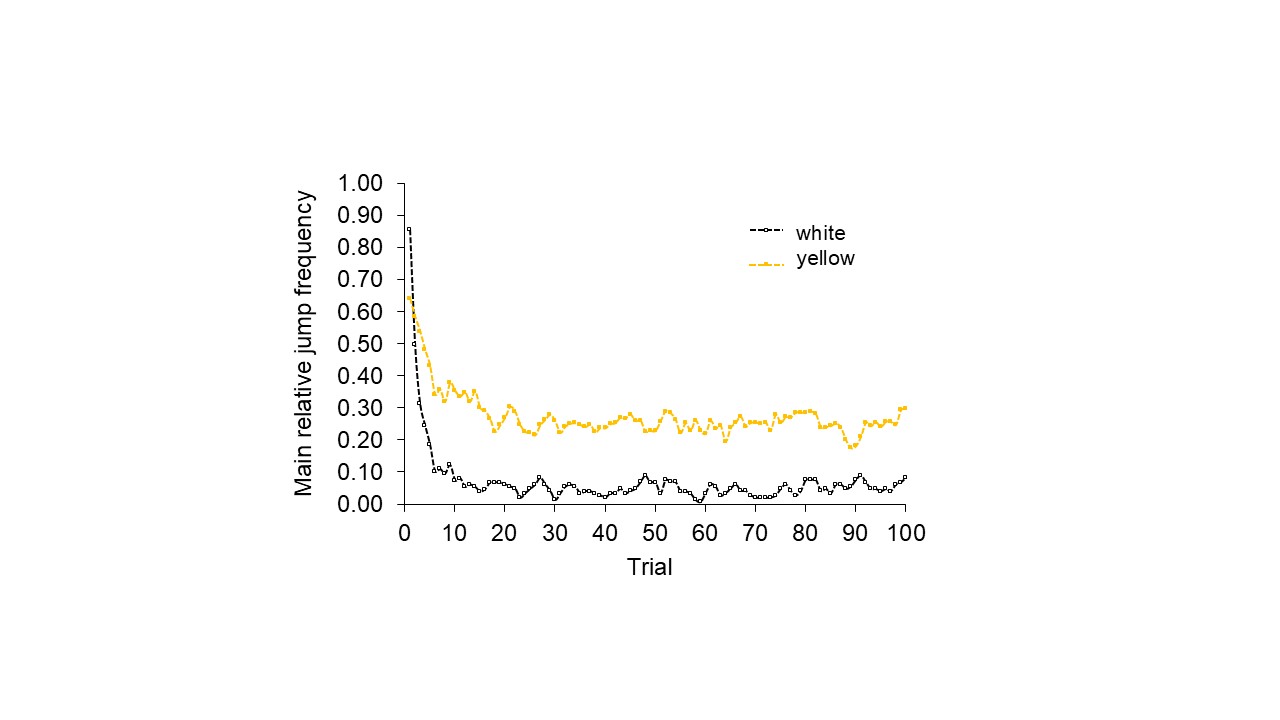


**Supplementary Figure 7:** **Habituation to series of light-off stimuli depends on the length of Inter Trial Intervals (ITI) separating the individual stimulus.** **(a)** Light-off jump reflex Habituation protocol. Series of frequent (e.g., 1s ITI) light-off stimuli results in habituation, i.e., waning of the flies’ initial jump response. **(b)** Habituation curve of white eyed (n=128) and yellow eyed (n=128) flies exposed to 100x repeats of light-off stimuli (1s ITI, 50🡪0 light units, 15ms duration). **(c)** Repeats of light-off stimuli at 5s ITI evokes non-habituating jump responses in white and yellow eyed flies (50🡪0 light units, 15ms duration). Ten Light-off stimuli repeats protocol were repeated 10 times for each fly and the jump responses were averaged for each repeat position (repeated on three or five different days; white-eyed: n=96; pale-yellow-eyed: n=160).


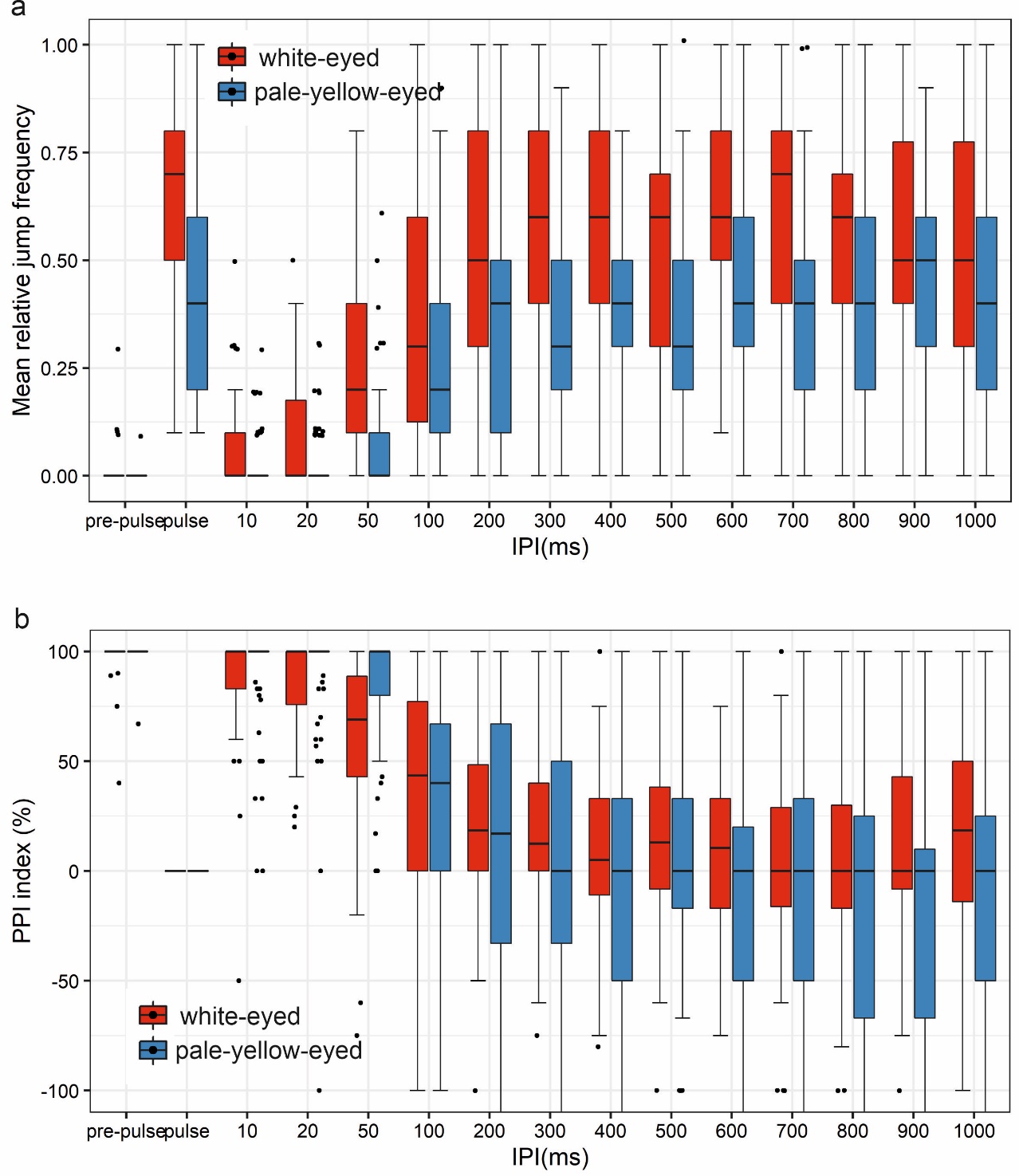


**Supplementary Figure 8: PPI results of white-eyed and pale-yellow-eyed flies in the range of 10 − 1000 ms IPIs**. **(a)** Mean Relative Jump Frequencies represented by box plots. Experiments were performed on two different days (n=64, each). **(b)** *Drosophila* PPI expressed in PPI indices. PPI indices are calculated from jump response data shown in **a** by the formula: 100 – ((PPI score/Pulse score) x100).


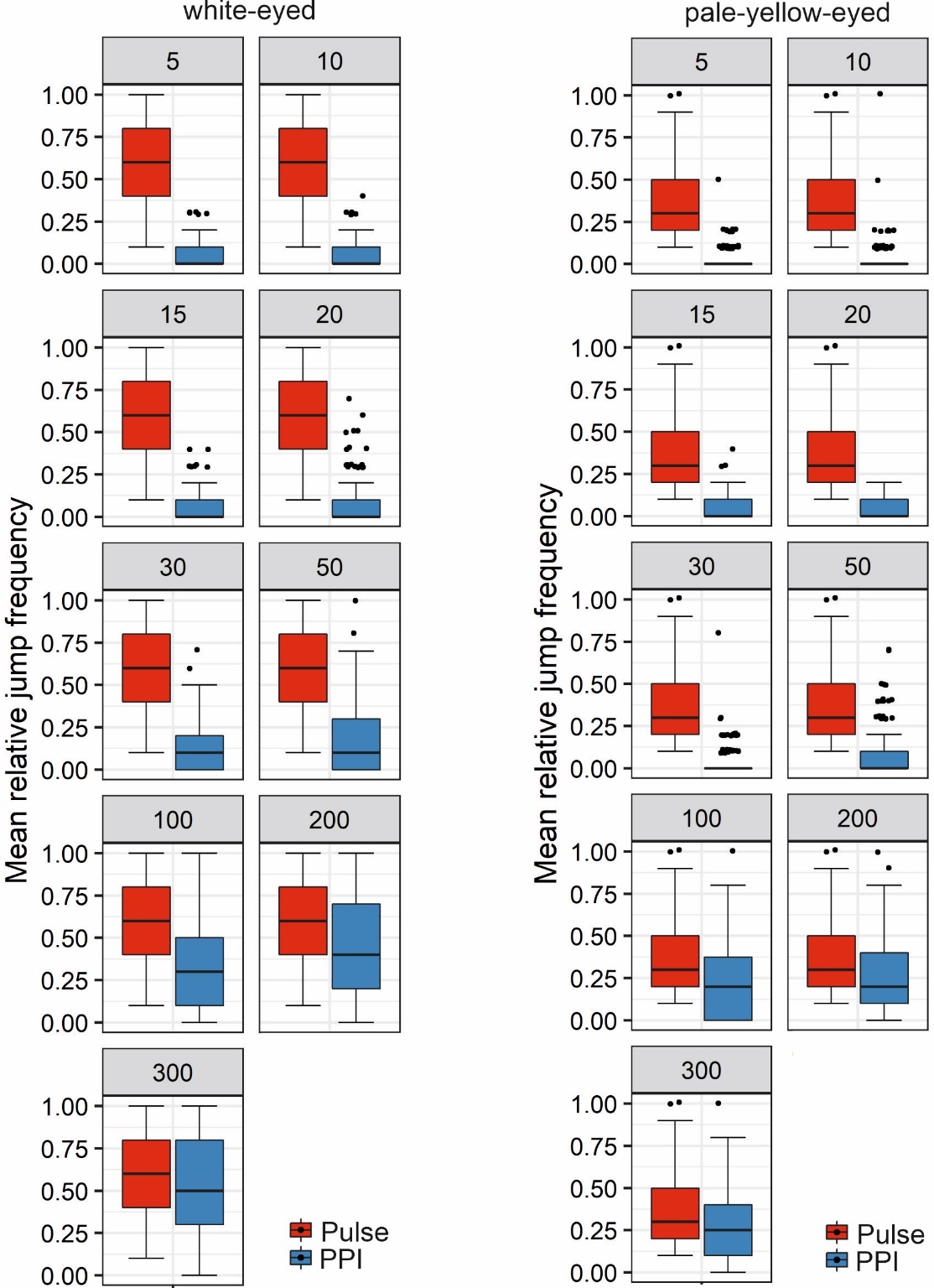
 white yellow

**Supplementary Figure 9: Pairwise comparison of white- and yellow eyed *Drosophila* males’ Mean Relative Jump Frequencies to Pulse and PPI stimuli at different IPIs.** Data is from Supplementary Figure 8**.**


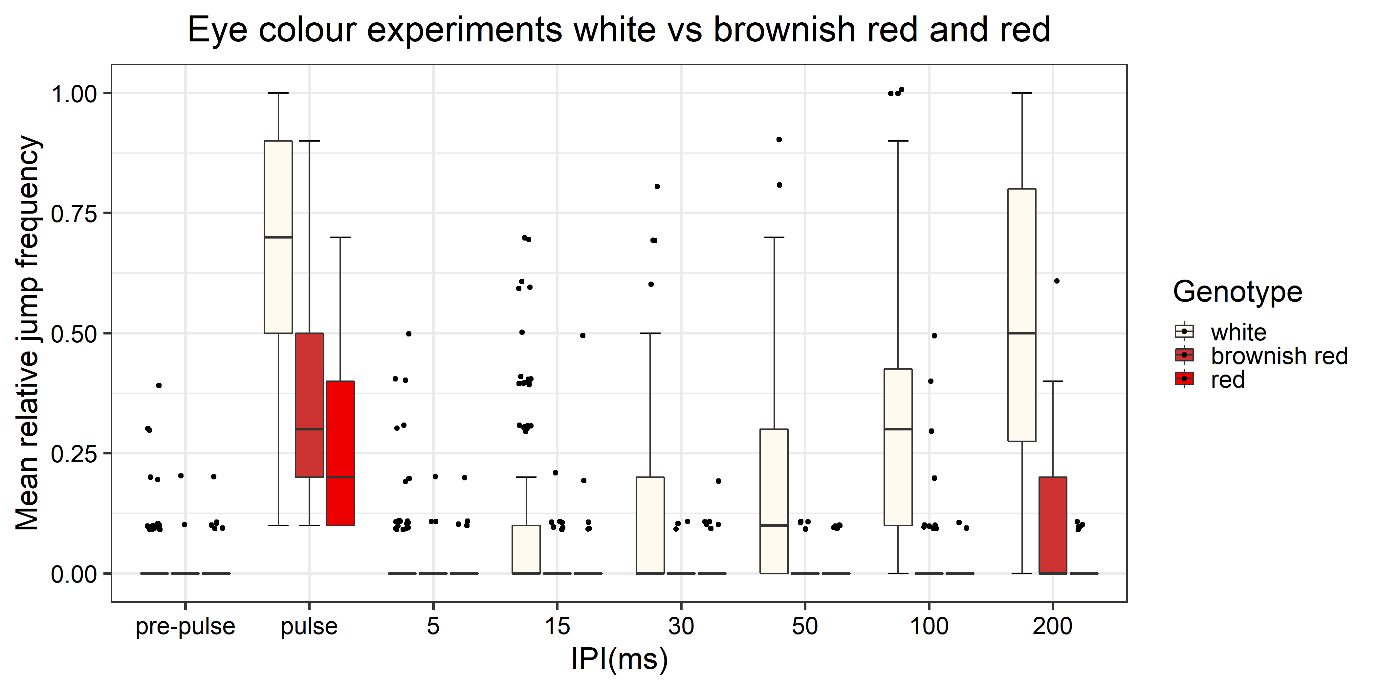
a


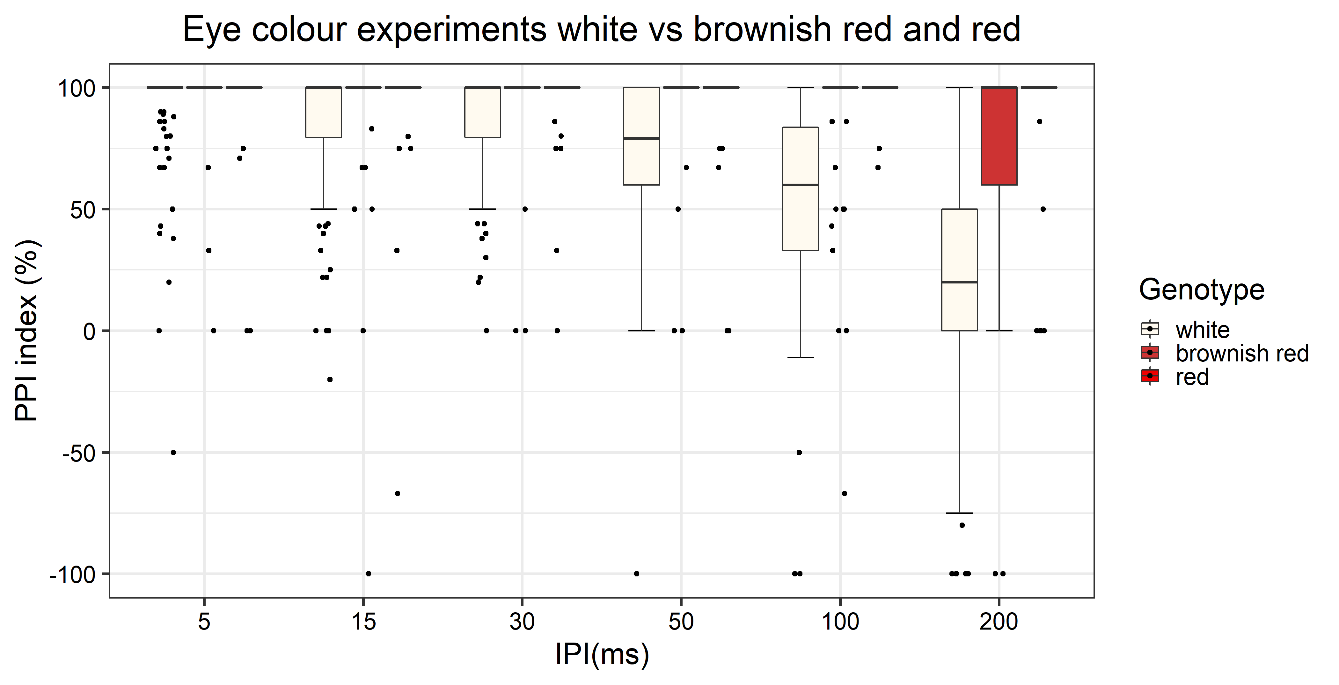


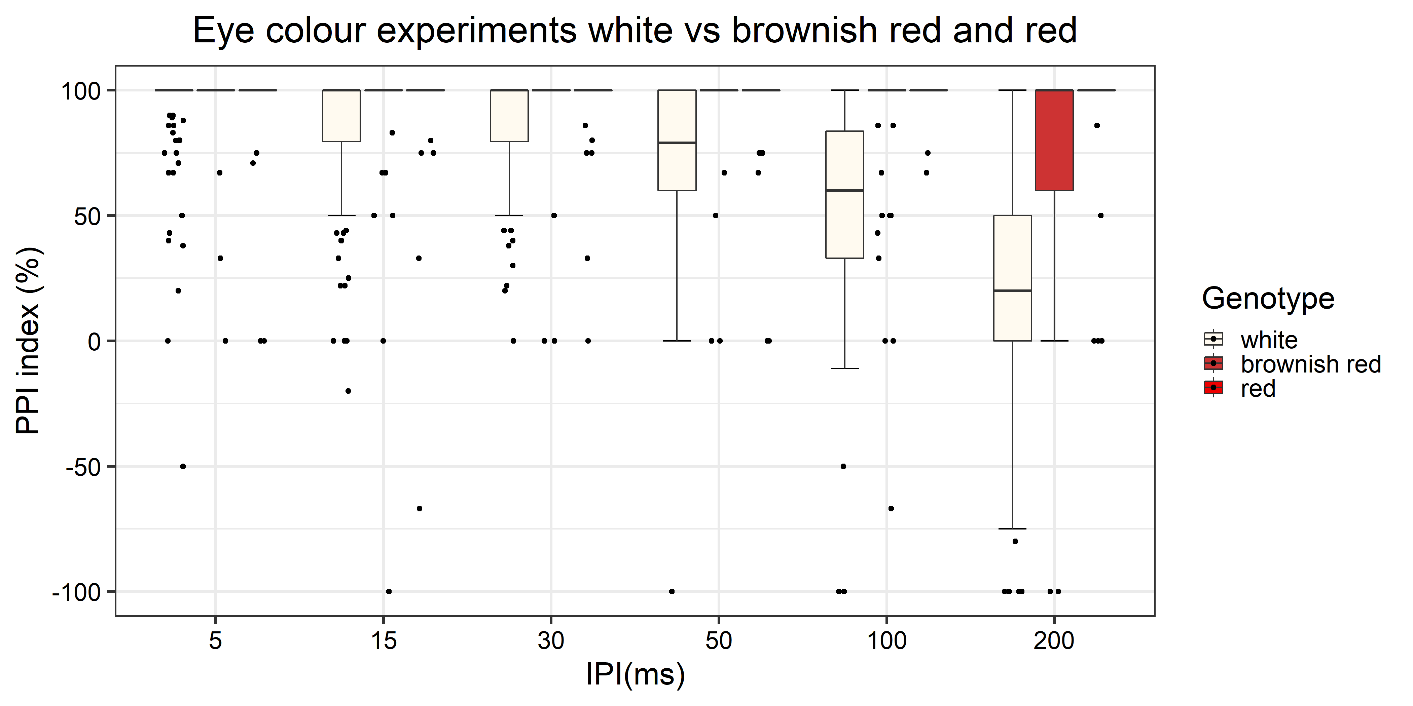
b


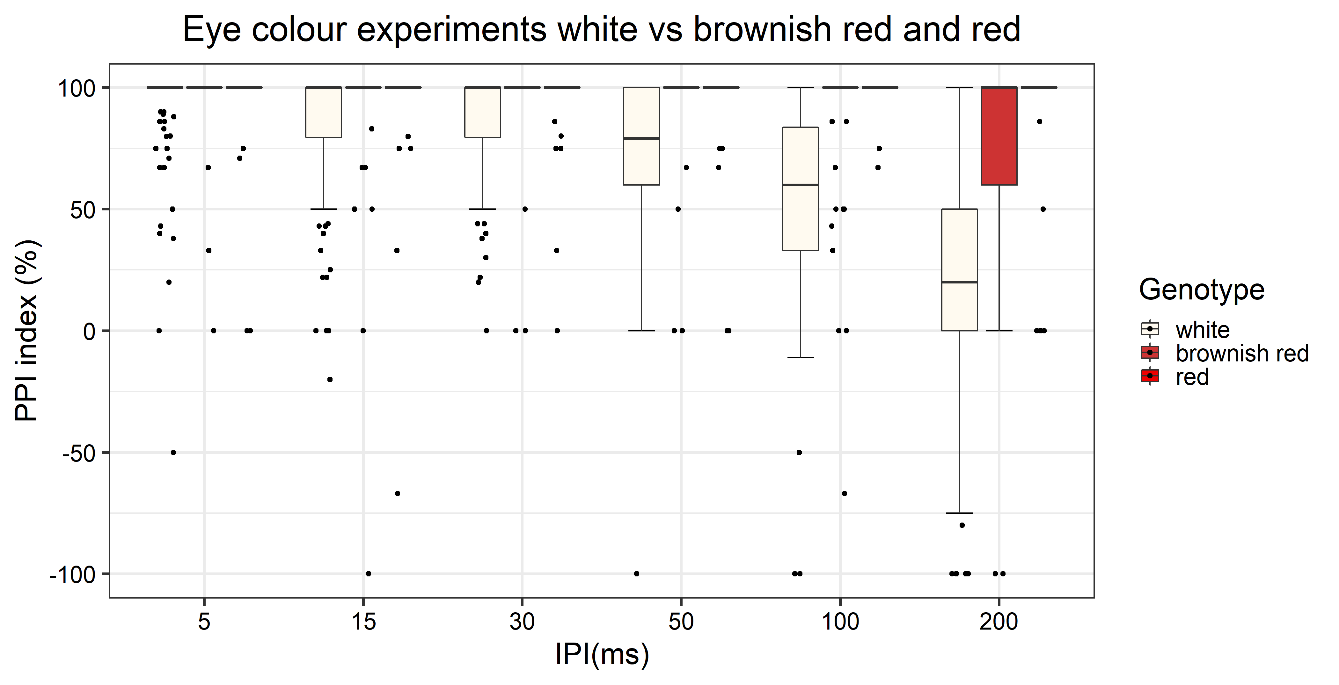


**Supplementary Figure 10:** **Weak jumping reaction to Pulse stimuli masks the normal PPI effect in flies with strong eye pigmentation**. **(a)** Mean Relative Jump Frequencies of white- (n=95), brownish red- (n=53) and red-eyed (n=48) flies in response to PPI light-dimming stimuli from 5 to 200 ms IPIs. Results are represented by box plots. Experiments were conducted on three different days. **(b)** Data from panel (a) shown as PPI indices. PPI indices were calculated from the jump response data using the formula: **100 – ((PPI score/Pulse score) x100).** For full genotypes, see Supplementary Table 1, for PPI indices see Supplementary Table 2.

**
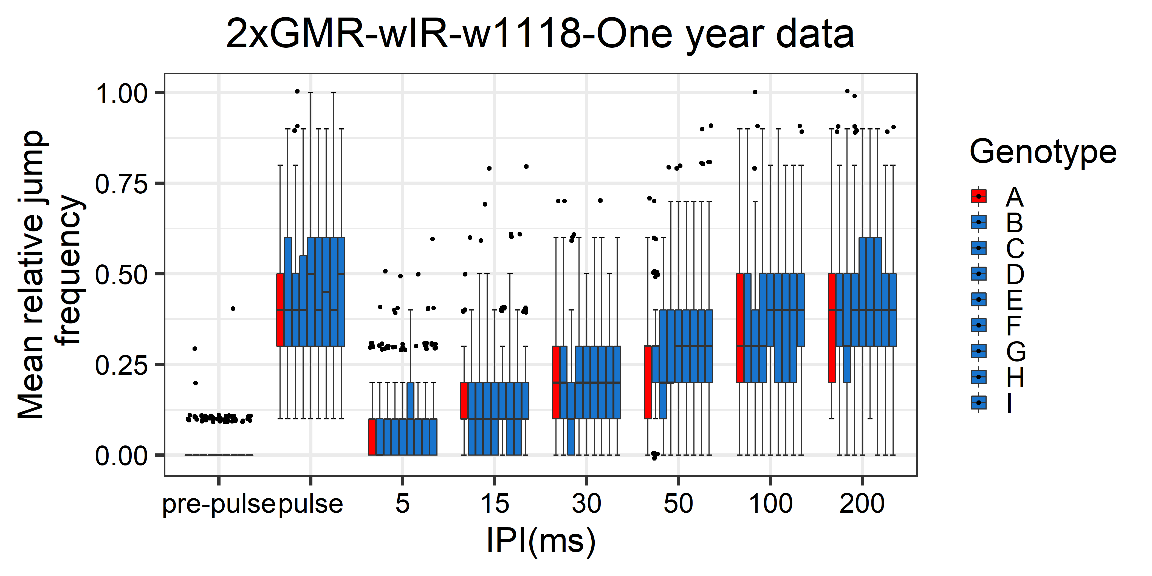
**a


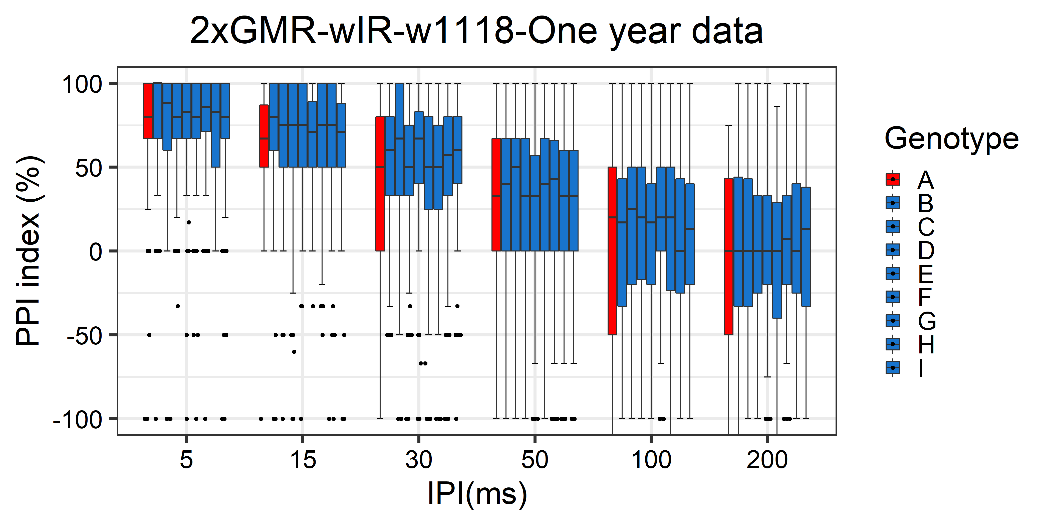
b


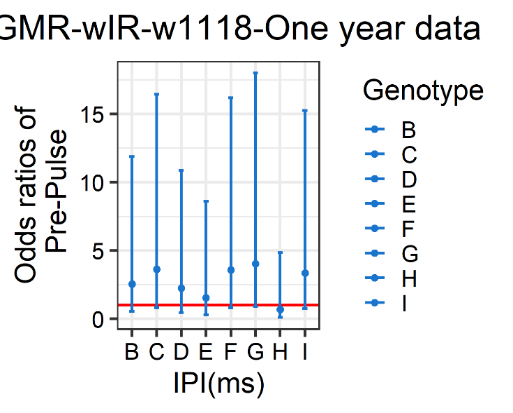
 d


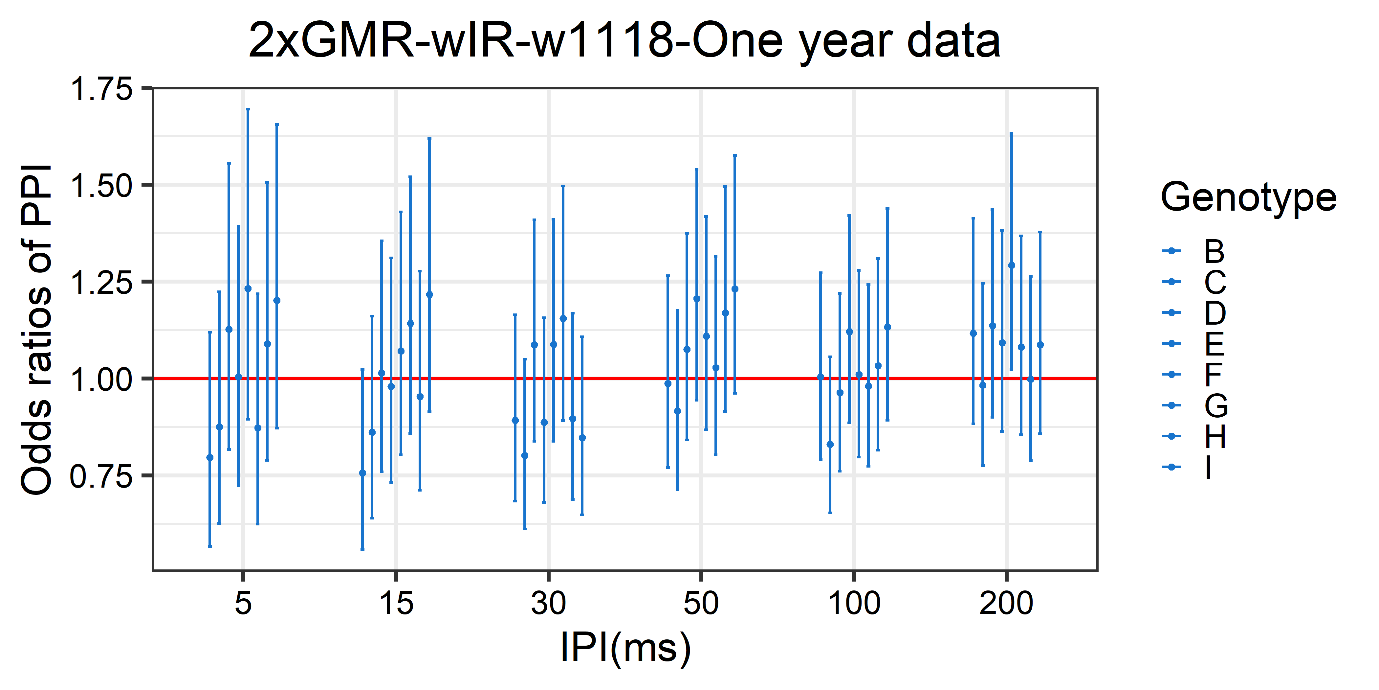
c


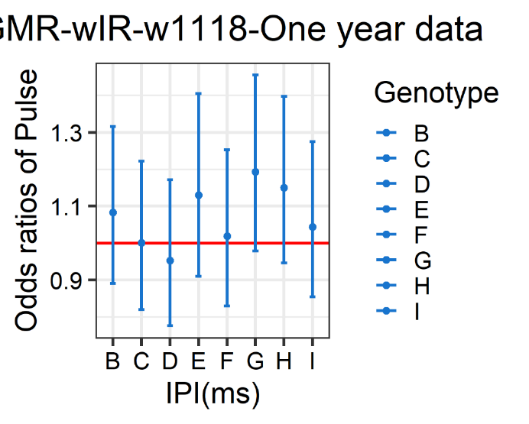


**Supplementary Figure 11: High reproducibility of data collected over a one-year period time** (white-eyed, 2XGMR-wIR). Data of 9 groups (n=128/group; 4 days, 32 flies/day) were compared to each other in the range of 5-200 ms IPI-s. For comparison, group No.1 (red) was chosen as control. **(a)** Mean Relative Jump Frequencies to Pre-Pulse, Pulse and PPI light dimming stimuli are represented by box plots. **(b)** Jump response data shown in **a** are expressed in PPI indices. **(c, d)** Jumping odds ratios following PPI, Pre-pulse as well as Pulse stimuli estimated by logistic regression model (GLMM) from data shown in **a**. Red horizontal line represents group No. 1


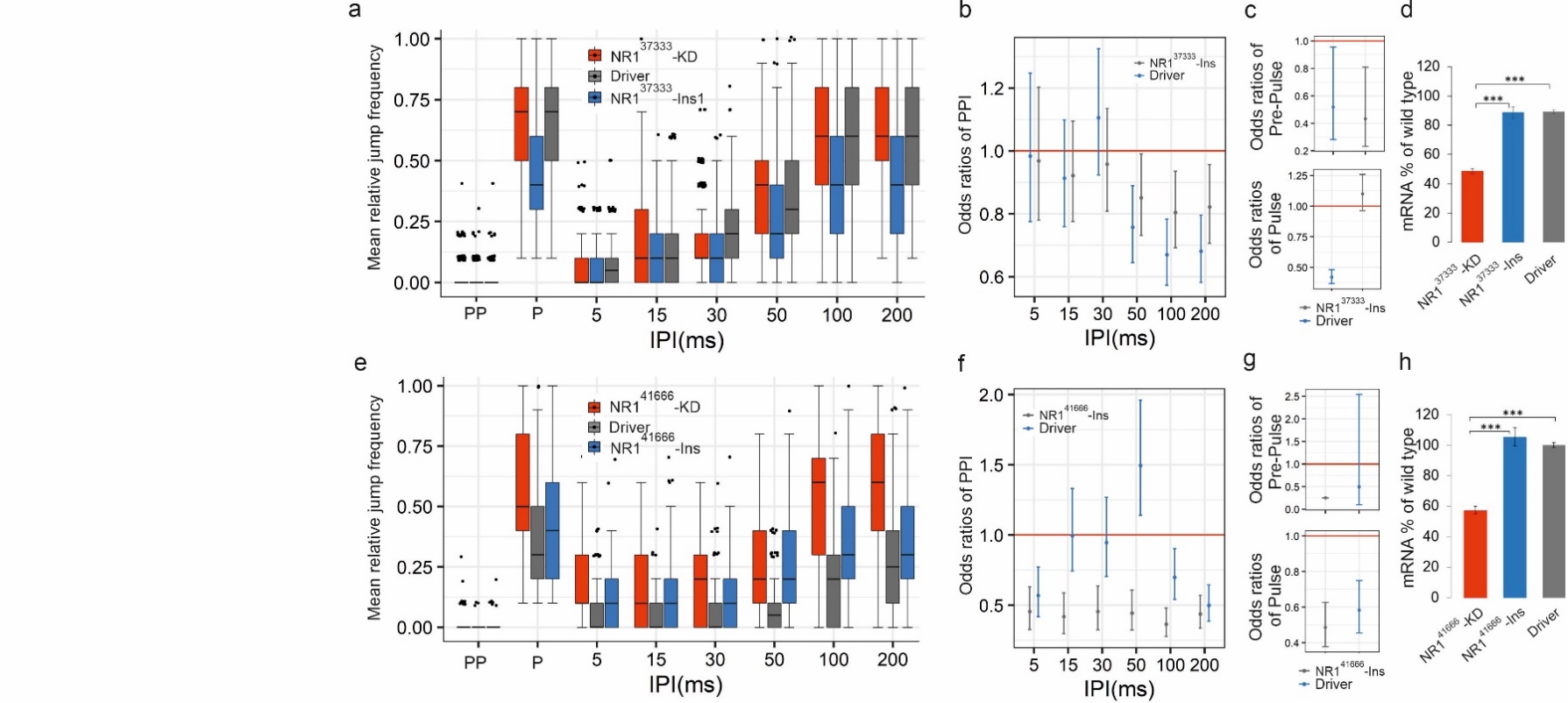


**Supplementary Figure 12: Knock-down mutants of schizophrenia susceptibility gene orthologue *Nmdar1* (NR1 in the charts) show PPI phenotype.** **(a** and **e)** Jump frequency of *Nmdar1* RNAi Knock-down flies (*NR1^37333^*-KD, n=350 and *NR1^41666^*-KD, n=125, respectively) compared to the appropriate control genotypes, UAS-NR1-RNAi inserts alone (*NR1^37333^*-Ins, n=336 and *NR1^41666^*-Ins, n=124, respectively) as well as *2xGMR-wIR*; *elav-Gal4,* *UAS-Dicer-2* and *elav-Gal4 ^c155^, GMR-wIR* (Driver, n=350 and n=118, respectively) alone in PPI experiments. For detailed genotypes see Supplementary Table 3. **(b, c, f, g)** Control/mutant jumping odds ratios (genotypes as in **a** and **e**) following PPI, Pre-Pulse, as well as Pulse stimuli estimated by GLMM. At Odds Ratio 1 the red horizontal line indicates equal odds of jumping for the mutant (*NR1^37333^*-KD or *NR1^41666^*-KD) value and a given control genotype. Odds ratios for the two control genotypes are calculated as control/mutant jumping odds separately. Therefore, for a given control genotype odds ratios below 1 indicate lower chance of jumping, and in the case of PPI stimuli, stronger PPI compared to the mutant. When the error bars representing the 95% confidence intervals do not overlap with the corresponding red horizontal line, it is considered to be a sign of statistically significant difference. **(d, h)** Relative levels of NR1 mRNA in different genotypes. ***: P< 0.001.


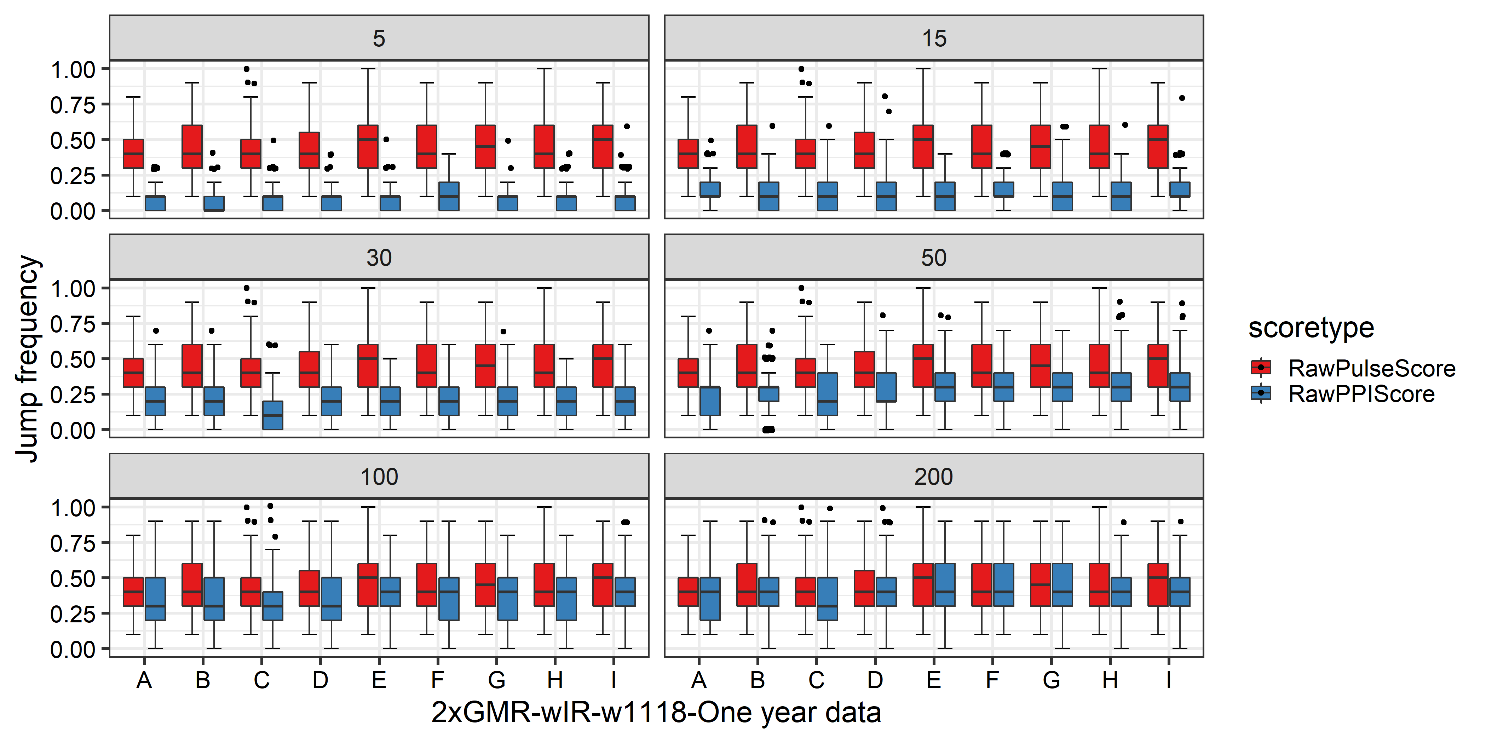
2xGMR-wIR

a

**Supplementary Figure 13 a:** **Pairwise visual comparison of Pulse and PPI jump frequencies of white-eyed, 2XGMR-wIR.** Plots were generated from data shown in Supplementary Fig.11.

**
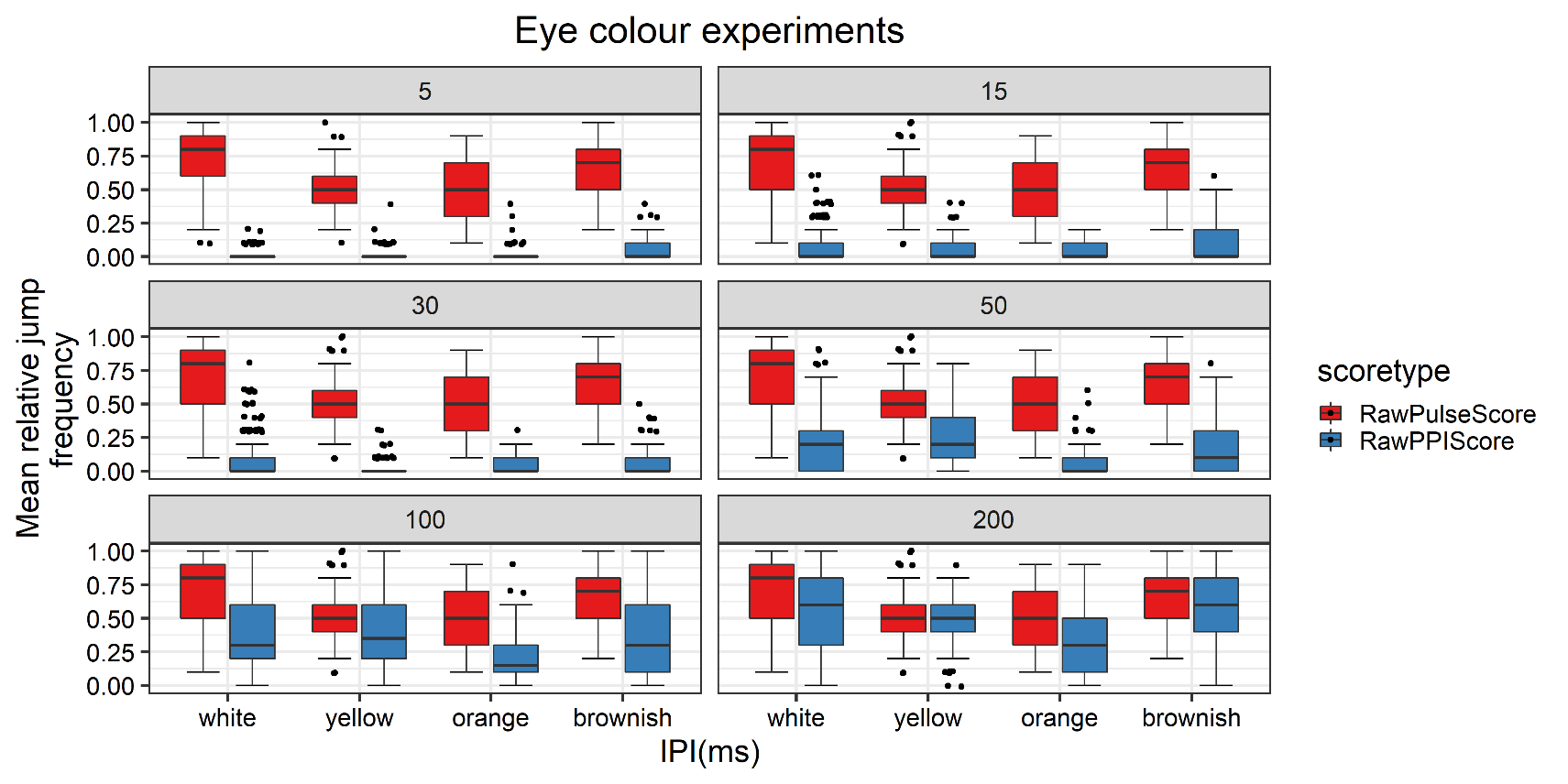
**

b


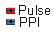


**Supplementary Figure 13 b:** **Pairwise visual comparison of Pulse and PPI jump frequencies of all genotypes tested.** Plots were generated from data shown in Fig. 1g.


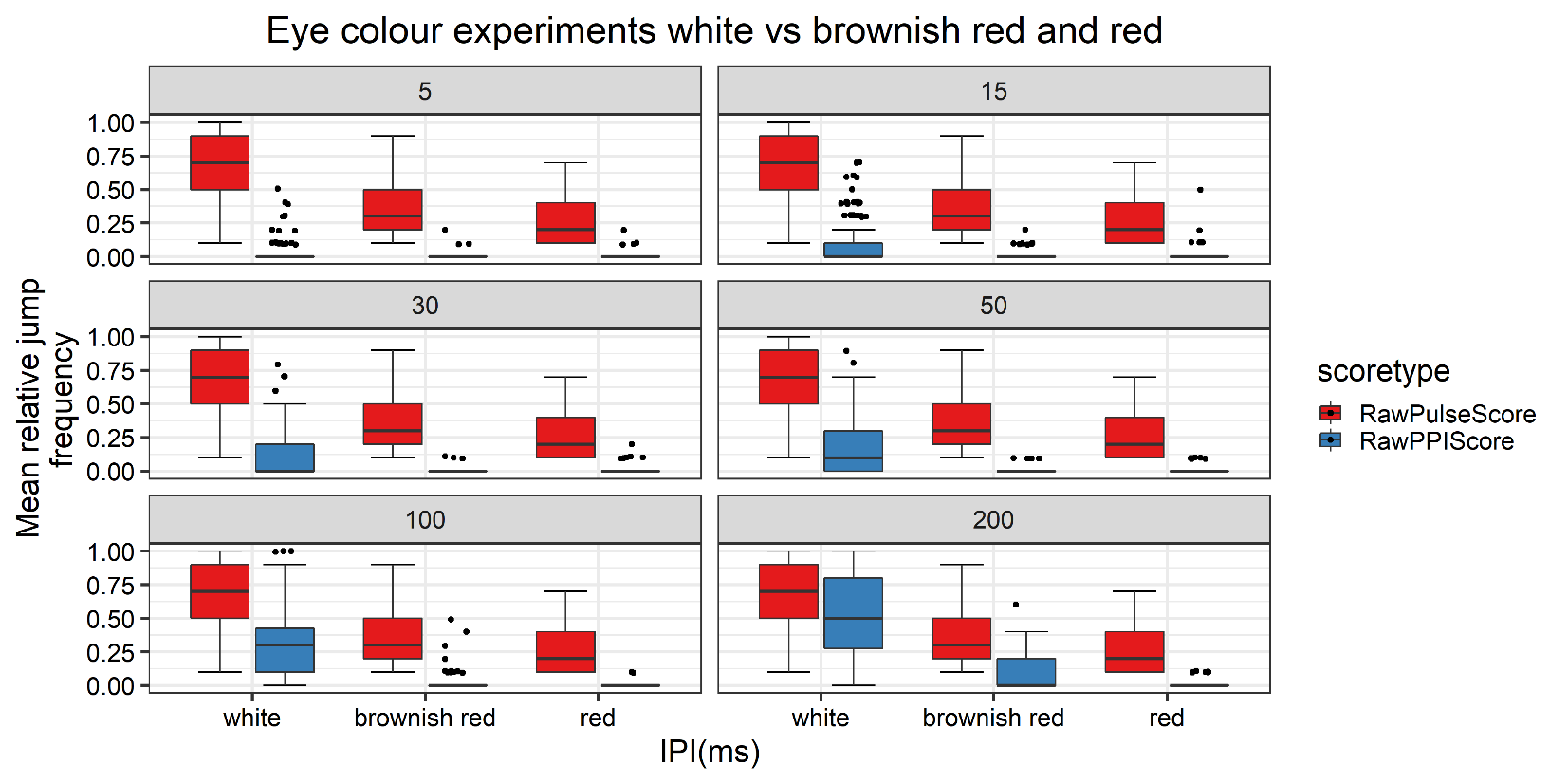
c


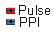


**Supplementary Figure 13 c:** **Pairwise visual comparison of Pulse and PPI jump frequencies of all genotypes tested.** Plots were generated from data shown in Supplementary Fig. 10a.


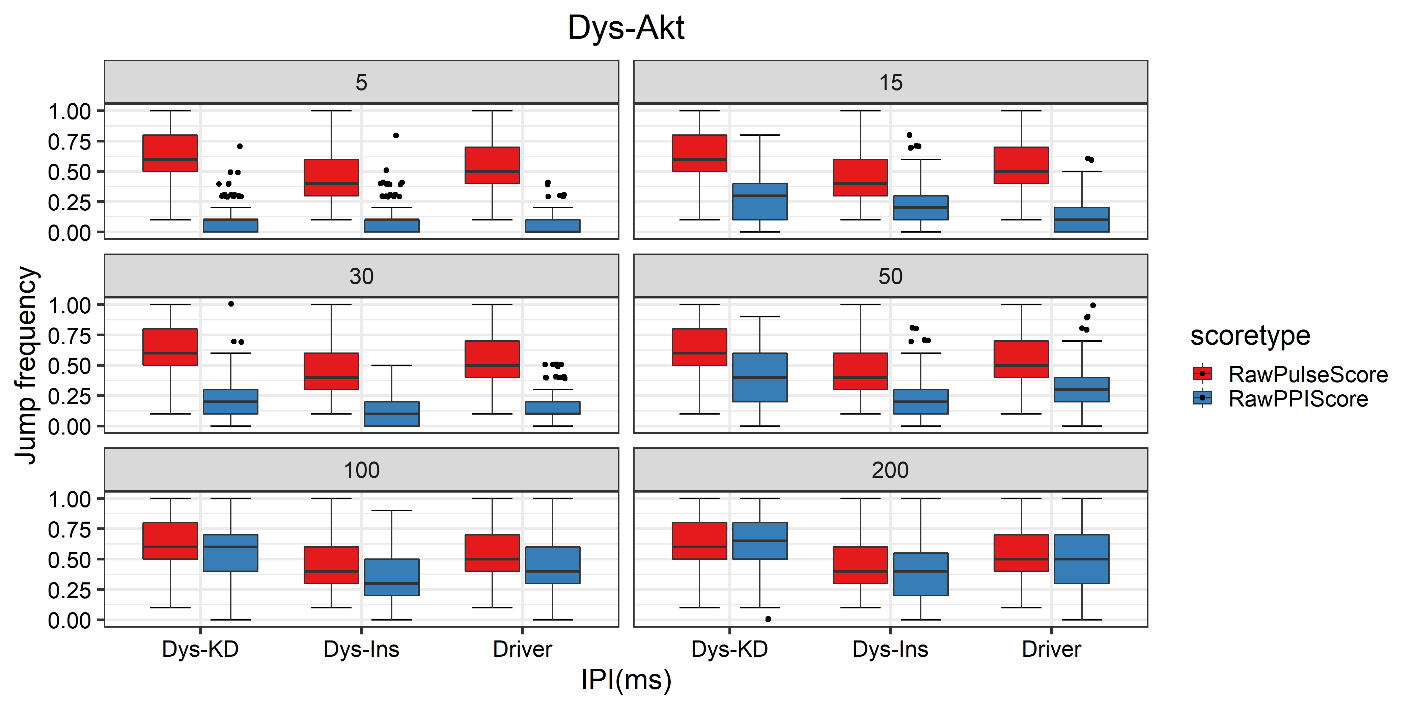
d

**Supplementary Figure 13 d:** **Pairwise visual comparison of Pulse and PPI jump frequencies of all genotypes tested.** Plots were generated from data shown in Fig. 3a.


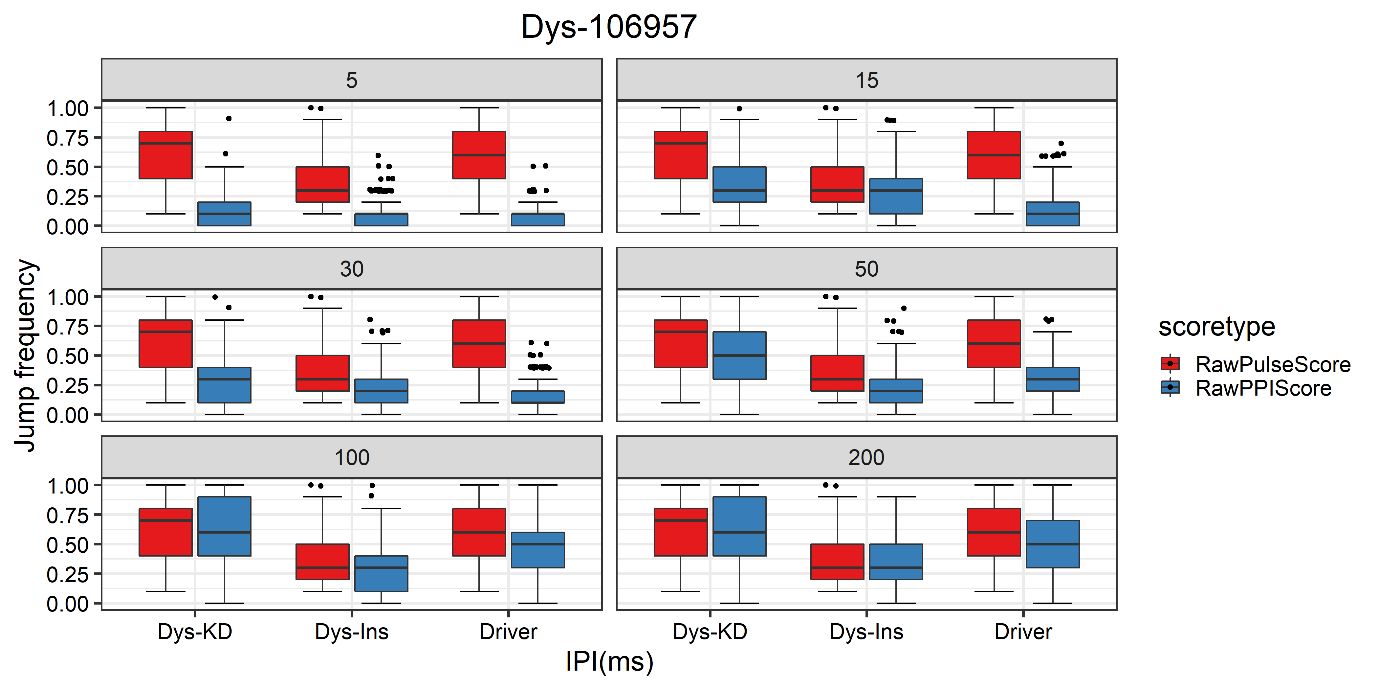


e

**
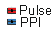
**

**Supplementary Figure 13 e:** **Pairwise visual comparison of Pulse and PPI jump frequencies of all genotypes tested.** Plots were generated from data shown in Fig. 3e.


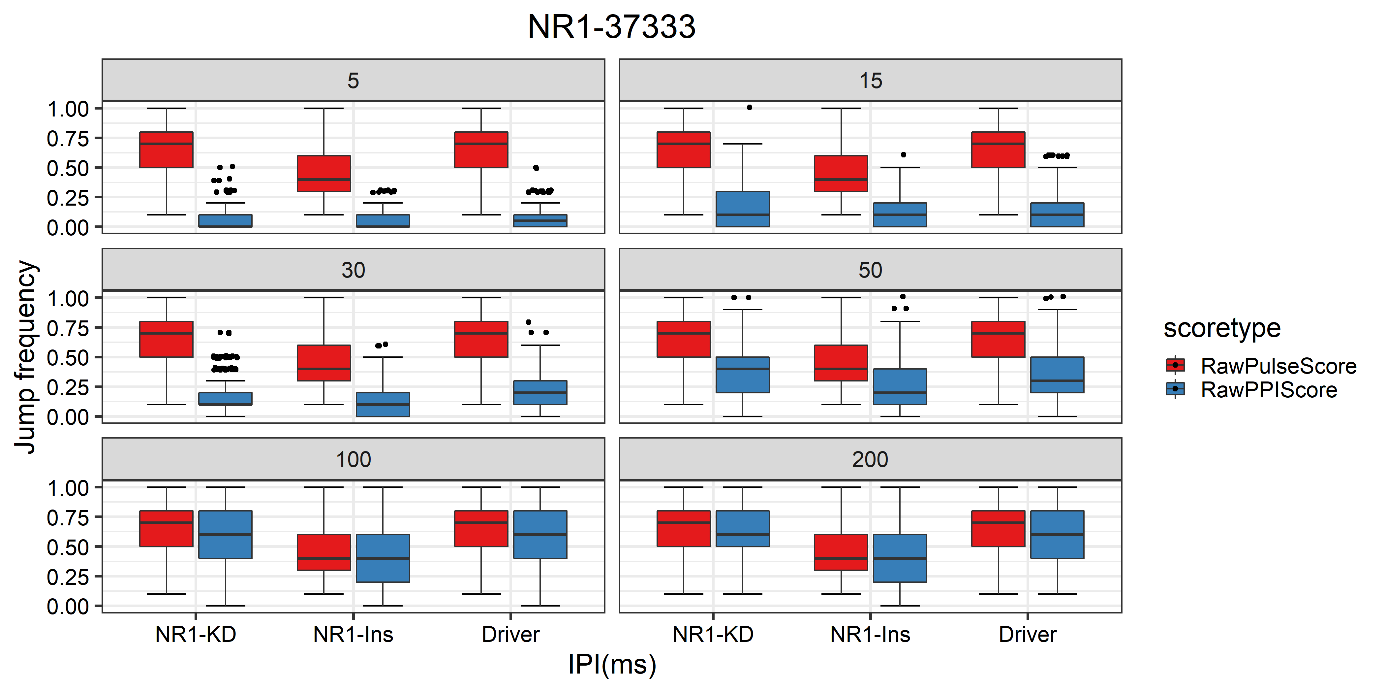
f

**Supplementary Figure 13 f:** **Pairwise visual comparison of Pulse and PPI jump frequencies of all genotypes tested.** Plots were generated from data shown in Supplementary Fig. 12a.


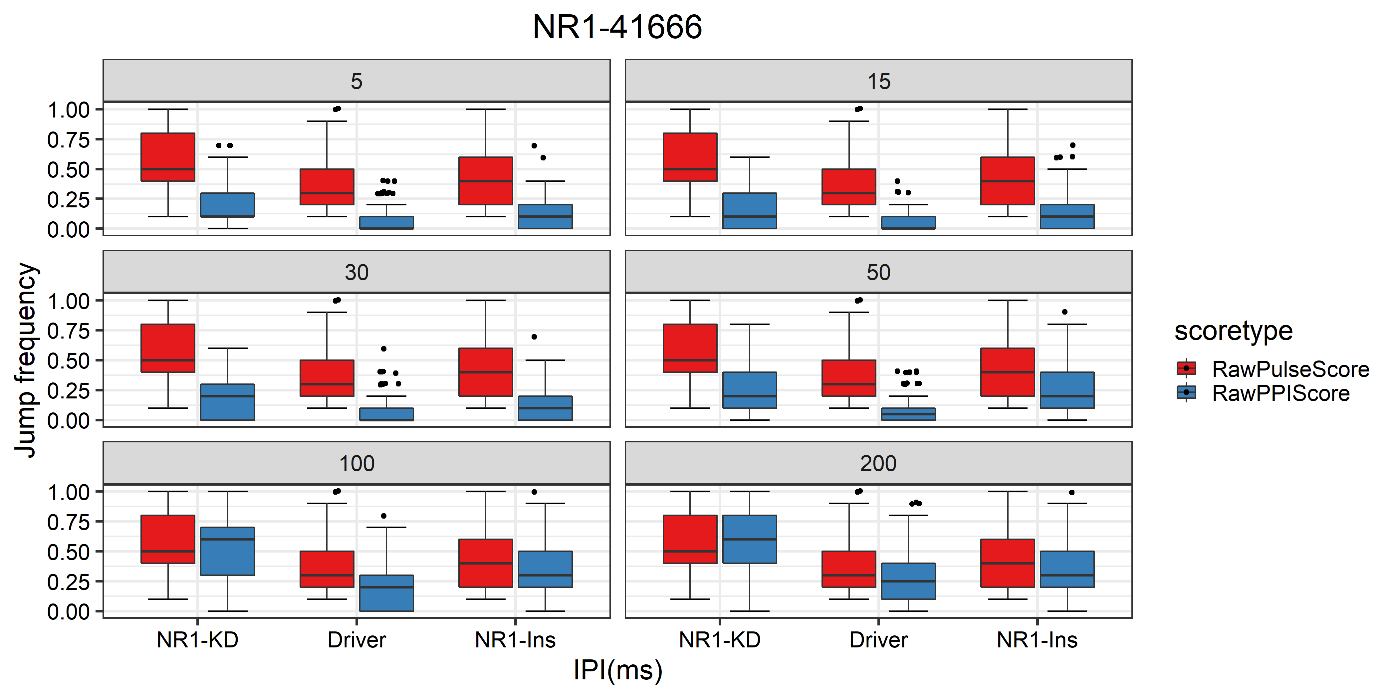
g

**Supplementary Figure 13 g:** **Pairwise visual comparison of Pulse and PPI jump frequencies of all genotypes tested.** Plots were generated from data shown in Supplementary Fig. 12e.

NR1-41666 and NR1-Del

**
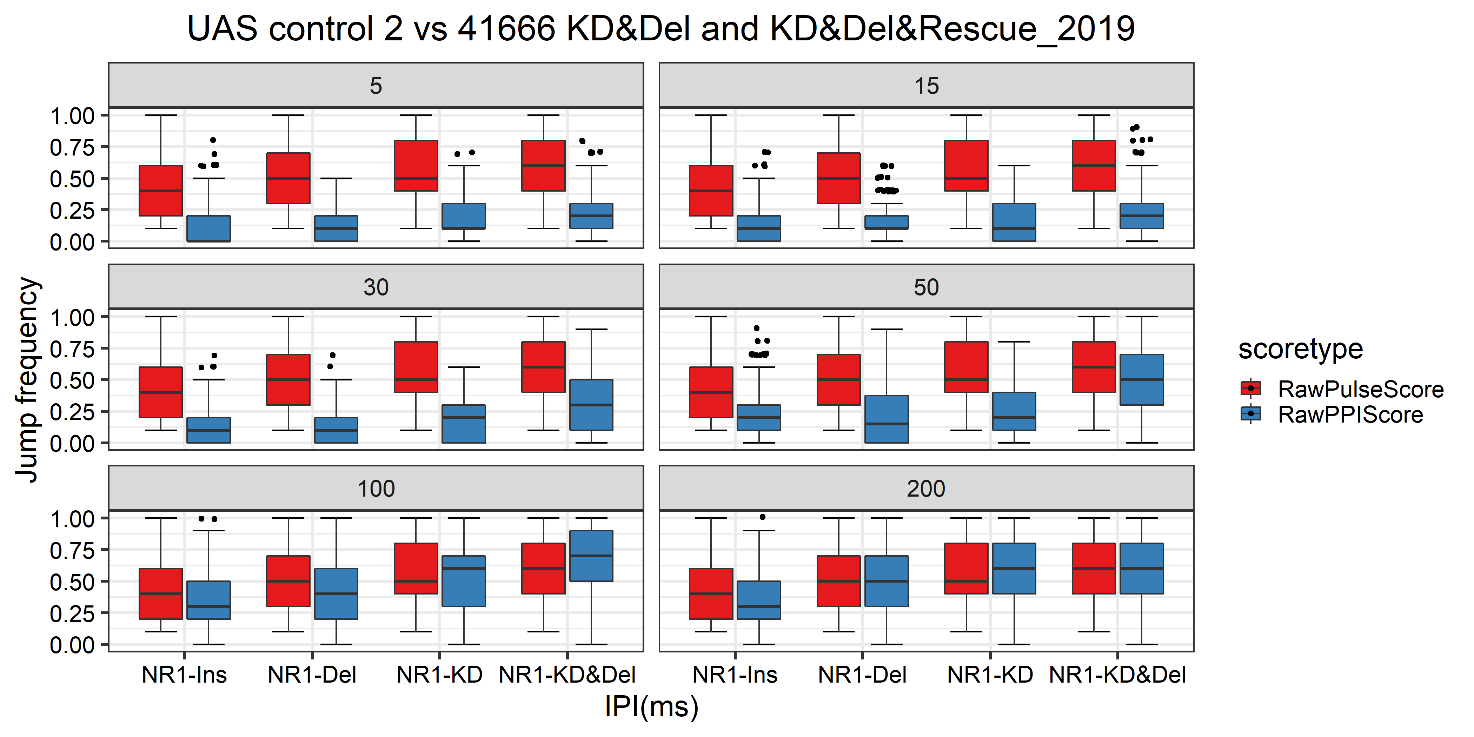
**h

**Supplementary Figure 13 h:** **Pairwise visual comparison of Pulse and PPI jump frequencies of all genotypes tested.** Plots were generated from data shown in Fig. 4a.


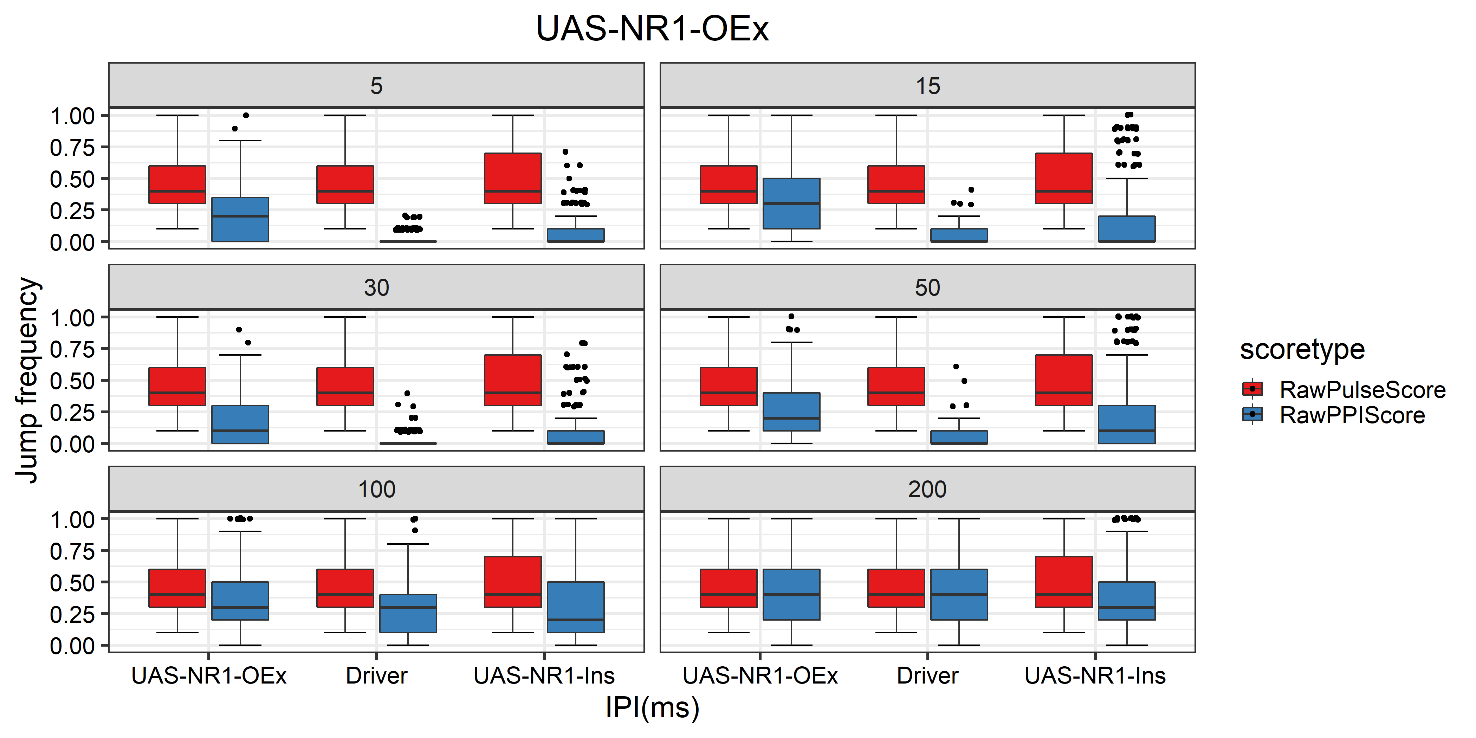
i

**Supplementary Figure 13 i:** **Pairwise visual comparison of Pulse and PPI jump frequencies of all genotypes tested.** Plots were generated from data shown in Fig. 5a.

| Abbreviation used in the text | Detailed genotype |  |
| --- | --- | --- |
|  |  |  |
| White-eyed | *w^1118^ Iso31/Y* |  |
| Yellow-eyed | *w^+^, GMR-wIR/Y* |  |
| Orange-eyed | *w^-^/Y; +; UAS-TIR1/+* |  |
| Brownish-eyed | *w^-^/Y; UAS-TIR1/+; +* |  |
| Brownish red-eyed | *w^-^/Y; +; UAS-Nmdar1* |  |
| Red-eyed | *w^+^/Y, Canton-S* |  |
| *2xGMR-wIR* | *w^+^/Y; 2xGMR-wIR-3/2-25/1/+* |  |
| Driver (Fig.5; Supplementary Fig. 9) | *w^-^, elav-Gal4 ^c155^, GMR-wIR/Y* |  |
| Driver (Fig.3; Supplementary Fig. 9) | *w^-^/Y; 2xGMR-wIR-3/2-25/1/+; elav-Gal4, UAS-Dicer-2/+* |  |
| *Dysb^Akt^*-Ins | *w^+^, GMR-wIR/Y; +; UAS-Dysbindin-RNAi^Akt^/+* |  |
| *Dysb^Akt^*-KD | *w^-^/Y; 2xGMR-wIR-3/2-25/1/+; elav-Gal4, UAS-Dicer-2 / UAS-Dysbindin-RNAi^Akt^* |  |
| *Dysb^106957^*-Ins | *w^+^, GMR-wIR/Y; +; UAS-Dysbindin-RNAi^106957^/+* |  |
| *Dysb^106957^*-KD | *w^-^/Y; 2xGMR-wIR-3/2-25/1/+; elav-Gal4, UAS-Dicer-2 / UAS-Dysbindin-RNAi^106957^* |  |
| *NR1^37333^*-Ins | *w^+^, GMR-wIR /Y; +; UAS-Nmdar1-RNAi^37333^/+* |  |
| *NR1^37333^-KD* | *w^-^/Y; 2xGMR-wIR-3/2-25/1/+; elav-Gal4, UAS-Dicer-2 / UAS-Nmdar1-RNAi^37333^* |  |
| *NR1^41666^*-Ins | *w^+^, GMR-wIR /Y; +; UAS-Nmdar1-RNAi^41666^/+* |  |
| *NR1^41666^-KD* | *w^-^, elav-Gal4^c155^, GMR-wIR/Y; +; UAS-Nmdar1-RNAi^41666^/+* |  |
| *NR1*-Del | *w^1118^; +; BSC179/+* |  |
| *NR1*-KD&Del | *w^-^, elav-Gal4^c155^, GMR-wIR/Y; +; UAS-Nmdar1-RNAi^41666^/BSC179* |  |
| *UAS-NR1* | *w^1118^/Y; +; UAS-Nmdar1/+* |  |
| *elav-UAS-NR1* | *w^-^, elav-Gal4^c155^, GMR-wIR/Y; +; UAS-Nmdar1/+* |  |

**Supplementary Table 1:** **Genotypes of tested flies.**

**Supplementary Table 2:** **PPI parameter settings and PPI indices for flies with different eye colours.**

| **P values of GLMM analysis** | | | | | | | | | |
| --- | --- | --- | --- | --- | --- | --- | --- | --- | --- |
| *Dysb^Akt^*-KD (n=242) | | | | | | | | | |
|  |  |  | IPI | | | | | | |
|  | Pre-Pulse | Pulse | 5 | 15 | 30 | 50 | 100 | 200 |  |
| *Dysb^Akt^*-Ins (n=235) | 0.4314 | <10-4 | 0.1099 | 0.72 | 0.0001 | <10-4 | <10-4 | <10-4 |  |
| Driver (n=248) | 0.0231 | <10-4 | 0.0016 | <10-4 | 0.0001 | 0.0037 | 0.0013 | 0.0003 |  |
|  |  |  |  |  |  |  |  |  |  |
| *Dysb^106957^*-KD (n=248) | | | | | | | | | |
|  |  |  | IPI | | | | | | |
|  | Pre-Pulse | Pulse | 5 | 15 | 30 | 50 | 100 | 200 |  |
| *Dysb^106957^*-Ins (n=230) | 0.2457 | <10-4 | 0.6553 | 0.0038 | 0.1155 | <10-4 | <10-4 | <10-4 |  |
| Driver (n=253) | 0.4627 | 0.0029 | <10-4 | <10-4 | <10-4 | <10-4 | <10-4 | 0.0005 |  |
|  |  |  |  |  |  |  |  |  |  |
| *NR1^37333^*-KD (n=350) | | | | | | | | | |
|  |  |  | IPI | | | | | | |
|  | Pre-Pulse | Pulse | 5 | 15 | 30 | 50 | 100 | 200 |  |
| *NR1^37333^*-Ins (n=336) | 0.0353 | <10-4 | 0.8914 | 0.3366 | 0.274 | 0.0007 | <10-4 | <10-4 |  |
| Driver (n=350) | 0.0088 | 0.1608 | 0.7713 | 0.3542 | 0.6192 | 0.0372 | 0.0047 | 0.0115 |  |
|  |  |  |  |  |  |  |  |  |  |
| *NR1^41666^*-KD (n=125) | | | | | | | | | |
|  |  |  | IPI | | | | | | |
|  | Pre-Pulse | Pulse | 5 | 15 | 30 | 50 | 100 | 200 |  |
| *NR1^41666^*-Ins (n=124) | 0.4021 | <10-4 | 0.0003 | 0.9738 | 0.7104 | 0.0037 | 0.006 | <10-4 |  |
| Driver (n=118) | <10-4 | <10-4 | <10-4 | <10-4 | <10-4 | <10-4 | <10-4 | <10-4 |  |
|  |  |  |  |  |  |  |  |  |  |
| *NR1^41666^*-Ins (n=293) | | | | | | | | | |
|  |  |  | IPI | | | | | | |
|  | Pre-Pulse | Pulse | 5 | 15 | 30 | 50 | 100 | 200 |  |
| NR1-Del (n=258) | 0.2527 | <10-4 | 0.5055 | 0.4037 | 0.006 | 0.0006 | 0.1556 | 0.0015 |  |
| NR1-KD (n=125) | 0.7972 | 0.0006 | <10-4 | 0.5907 | 0.5421 | 0.0023 | 0.0015 | <10-4 |  |
| NR1-KD&Del (n=253) | <10-4 | <10-4 | <10-4 | 0.0002 | <10-4 | <10-4 | <10-4 | <10-4 |  |
|  |  |  |  |  |  |  |  |  |  |
| *UAS-NR1*-OEx (n=175) | | | | | | | | | |
|  |  |  | IPI | | | | | | |
|  | Pre-Pulse | Pulse | 5 | 15 | 30 | 50 | 100 | 200 |  |
| *UAS-NR1*-Ins (n=177) | <10-4 | 0.1823 | <10-4 | <10-4 | <10-4 | 0.0001 | 0.0003 | 0.0009 |  |
| Driver (n=161) | 0.0001 | 0.0339 | <10-4 | <10-4 | <10-4 | <10-4 | 0.0036 | 0.9279 |  |

**Supplementary Table 3:** **Statistical comparison of jump frequencies between the indicated genotypes.** P values were calculated in GLMM with glmer function (mutant to controls or control to mutants as appropriate).

|  | *Dysb* mRNA % of wild type | SD % Up | | SD % Down | P values |
| --- | --- | --- | --- | --- | --- |
| Dysb^Akt^-KD | 64 | 4.1 | 3.9 | |  |
| Dysb^Akt^-Ins | 80 | 7.5 | 6.9 | | 0.0103 |
| Driver | 88 | 5.1 | 4.8 | | 0.0028 |
|  |  |  |  | |  |
|  |  |  |  | |  |
|  | *Dysb* mRNA % of wild type | SD % Up | SD % Down | | P values |
| Dysb^106957^-KD | 65 | 1.9 | 1.8 | |  |
| Dysb^106957^-Ins | 112 | 12.6 | 11.3 | | 0.0013 |
| Driver | 88 | 5.1 | 4.8 | | 0.0045 |
|  |  |  |  | |  |
|  |  |  |  | |  |
|  | NR1 mRNA % of wild type | SD % Up | SD % Down | | P values |
| NR1^37333^-KD | 49 | 1.9 | 1.9 | |  |
| NR1^37333^-Ins | 89 | 4.1 | 3.9 | | 0.00001 |
| Driver | 89 | 1.2 | 1.2 | | 0.000001 |
|  |  |  |  | |  |
|  |  |  |  | |  |
|  | NR1 mRNA % of wild type | SD % Up | SD % Down | | P values |
| NR1^41666^-KD | 58 | 1.6 | 1.6 | |  |
| NR1^41666^-Ins | 89 | 3.3 | 3.2 | | 0.0001 |
| Driver | 89 | 1.2 | 1.2 | | 0.00001 |
|  |  |  |  | |  |
|  |  |  |  | |  |
|  | NR1 mRNA % of wild type | SD % Up | SD % Down | | P values |
| NR1^41666^-Ins | 87 | 5.7 | 5.3 | |  |
| NR1-Del | 71 | 8.2 | 7.4 | | 0.0256 |
| NR1-KD | 54 | 6 | 5.4 | | 0.0006 |
| NR1-KD&Del | 37 | 1.6 | 1.5 | | 0.000002 |
|  |  |  |  | |  |
|  | NR1 mRNA % of wild type | SD % Up | SD % Down | | P values |
| UAS-NR1 | 106 | 6.9 | 6.5 | | 0.0000003 |
| elav-UAS-NR1 | 963 | 24.5 | 23.9 | |  |
| elav-ISO31 | 84 | 12.5 | 10.9 | | 0.00003 |

**Supplementary Table 4:** **Statistical comparison of *Dysbindin* (*Dysb*) or *Nmdar1* (NR1) mRNA levels between the indicated genotypes.** For P value calculation normalized Ct (ΔCt) values were used in Student’s t-test (two-tailed, unequal variance), n=4.

| **symbol** | **ref seq ID** | **FlyBase ID** | **description** | **Forward primer** | **Reverse primer** |
| --- | --- | --- | --- | --- | --- |
| ***gt*** | NM_080310.3 | CG7952 | Drosophila melanogaster *giant* (*gt*), mRNA | cagttcttcgatctcaagactgat | ctgctggtggtgctggta |
| ***Mnf*** | NM_168443.3 | CG11799 | Drosophila melanogaster *forkhead* box K, transcript variant K (*FoxK*), mRNA | cgcccagtcacatggataa | ggtgctgattgcaacacaat |
| ***rap*** | NM_167013.2 | CG3000 | Drosophila melanogaster retina aberrant in pattern (*rap*), transcript variant B, mRNA | tggtcgtcgcagaacgta | acgcgctccacagatagac |
| ***Nmdar1*** | NM_169059.2 | CG2902 | *Drosophila melanogaster* N-methyl-D-aspartate receptor-1, mRNA | agtccgaaggtgatggc | acacgtcgctggtgtatc |
| ***Dysb*** | NM_140807.4 | CG6856 | *Drosophila melanogaster Dysbindin (Dysb),* *mRNA* | aagctgcagagggaacgac | ttgttgctggtggtttgaat |

**Supplementary Table 5:** **Primer list of reference and tested genes used in RT-qPCR experiments.** Gene symbols, reference sequence IDs, FlyBase IDs and short descriptions are included.

**Supplementary Methods**

**getColour_RGB_HSB_code**

var mypic="";

var hsbmode=1; // off-on

var rgbmode=1; // off-on

macro "init" {

mypic=getTitle();

width=getWidth();

height=getHeight();

run("Remove Overlay");

if(hsbmode) {

run("Duplicate...", "title=hsb ignore");

run("HSB (32-bit)");

setSlice(1);

for(iy=0;iy<height;iy++)

for(ix=0;ix<width;ix++) {

hue=getPixel(ix, iy);

hue=hue+0.5;

hue=hue-floor(hue);

hue=hue-0.5;

setPixel(ix, iy, hue);

} // new hue scale runs from -0.5 (cyan-bueish) to 0.5 (greenish-cyan)

}

selectWindow(mypic);

if(rgbmode) {

run("Duplicate...", "title=rgb ignore");

run("RGB Stack");

}

selectWindow(mypic);

setTool("oval");

}

macro "get_HSB_RGB" {

selectWindow(mypic);

getStatistics(area, mean, min, max, std);

setResult("Area", nResults, mean);

setResult("Name", nResults-1, mypic);

if(hsbmode) {

if(selectionType==1) { // oval

getSelectionBounds(x, y, width, height);

selectWindow("hsb");

makeOval(x, y, width, height);

}

else if(selectionType==0) { // rect

getSelectionBounds(x, y, width, height);

selectWindow("hsb");

makeRectangle(x, y, width, height);

}

else if(selectionType==2 || selectionType==3) { // 2=polygon, 3=freehand

getSelectionCoordinates(xpoints, ypoints);

xpoints[xpoints.length]=xpoints[0];

ypoints[ypoints.length]=ypoints[0];

selectWindow("hsb");

makeSelection("freehand", xpoints, ypoints);

}

else {

exit("unsupported selection mode");

}

setSlice(1);

getStatistics(area, mean, min, max, std);

setResult("Hue", nResults-1, mean);

setSlice(2);

getStatistics(area, mean, min, max, std);

setResult("Sat", nResults-1, mean);

setSlice(3);

getStatistics(area, mean, min, max, std);

setResult("Bright", nResults-1, mean);

setSlice(1);

}

if(rgbmode) {

if(selectionType==1) { // oval

getSelectionBounds(x, y, width, height);

selectWindow("rgb");

makeOval(x, y, width, height);

}

else if(selectionType==0) { // rect

getSelectionBounds(x, y, width, height);

selectWindow("rgb");

makeRectangle(x, y, width, height);

}

else if(selectionType==2 || selectionType==3) { // 2=polygon, 3=freehand

getSelectionCoordinates(xpoints, ypoints);

xpoints[xpoints.length]=xpoints[0];

ypoints[ypoints.length]=ypoints[0];

selectWindow("rgb");

makeSelection("freehand", xpoints, ypoints);

}

else {

exit("unsupported selection mode");

}

setSlice(1);

getStatistics(area, mean, min, max, std);

setResult("Red", nResults-1, mean);

setSlice(2);

getStatistics(area, mean, min, max, std);

setResult("Green", nResults-1, mean);

setSlice(3);

getStatistics(area, mean, min, max, std);

setResult("Blue", nResults-1, mean);

setSlice(1);

}

selectWindow(mypic);

Overlay.addSelection("blue", 1);

}

**Logistic regression analysis of PPI**

The effect of genotype on Relative Jump Frequencies was analysed with generalized linear mixed-effects model (GLMM) using ‘glmer’ function (with binomially distributed jump data and logit link functions) from the ‘lme4’ R package^64^.

We applied two types of models, in both of which the number of jumps within a trial follows a binomial distribution:

$$y_{i}\sim Binomial(n_{i},p_{i})$$

where *i* is the index of a given trial consisting of 10 stimuli, *y_i_* is the number of jumps as a response to the stimuli in a given trial ranging between 0 and 10, *n_i_* is the number of stimuli administered (10) and *p_i_* is the probability of jumping as a response to the stimuli. Values of *p_i_* were estimated by the models below. These are logistic regression models, meaning that *logit(p_i_)*, i.e., the log-odds of jumping is modelled as the linear combination of the predictors (e.g., Genotype). They are also mixed effect models, meaning that some of the predictors (‘fixed effects’, e.g., Genotype) are modelled as having a constant effect which is in itself of interest, while others (e.g., Fly ID) are modelled as random effects, with an average value of 0, and a variability that is not explained by the fixed effect predictors in the model, e.g., the variability from fly to fly, which follows a normal distribution around 0.

First type of model examined the effect of genotype on Relative Jump Frequency to Pulse or Pre-Pulse stimuli with genotype as a fixed effect. Fly ID, Day (testing day), System (behaviour system; usually two systems are used in parallel), Box (behaviour box; two boxes form a system), Chamber (behaviour chamber, houses an individual fly; 8 chambers are built in a box), Operator (experimenter) and Run number (32 experiments run simultaneously) were included as categorical random effects (Formula 1). The model estimates the difference between two genotypes as the difference in their log-odds of jumping, which is the same as the logarithm of their odds ratio. The odds of jumping are the probability of jumping divided by the probability of not jumping as a response to the given stimulus. The odds ratio for two genotypes is the ratio of their odds. An odds ratio of 1 means equal probability of jumping, >1 means higher probability of jumping for the genotype in the numerator.

Formula 1):

$$logit\left( p_{i} \right)= \beta_{0}+ \beta^{Genotype}\cdot{Genotype}_{i}+ \alpha_{k[i]}^{Run}+ \alpha_{l[i]}^{Chamber}+ \alpha_{m[i]}^{Operator}+ \alpha_{n[i]}^{Fly ID}$$

$$\alpha_{k}^{Run}\sim N(\alpha_{p\left[ k \right]}^{Day}, \sigma_{Run}^{2})$$

$$\alpha_{p}^{Day}\sim N(0, \sigma_{Day}^{2})$$

$$\alpha_{l}^{Chamber}\sim N(\alpha_{q\left[ l \right]}^{Box}, \sigma_{Chamber}^{2})$$

$$\alpha_{q}^{Box}\sim N(\alpha_{r\left[ q \right]}^{System}, \sigma_{Box}^{2})$$

$$\alpha_{q}^{System}\sim N(0, \sigma_{System}^{2})$$

$$\alpha_{m}^{Operator}\sim N(0, \sigma_{Operator}^{2})$$

$$\alpha_{n}^{Fly ID}\sim N(0, \sigma_{Fly ID}^{2})$$

The other model examined the effect of genotype on PPI, i.e., suppression of Relative Jump Frequency to Pulse stimuli when these are preceded by Pre-Pulse stimuli at all tested IPIs (IPI: 5, 15, 30, 50, 100, 200, 300 ms). Genotype, IPI, jump response to Pulse (natural logarithm of the Relative Jump Frequency plus 0.1 to avoid taking the logarithm of 0), interaction between jump response to Pulse stimuli and IPI, and interaction between genotype and IPI were treated as fixed effects. Fly ID, Day, System, Box, Chamber, Operator and Run were treated as random effects (Formula 2). For the categorical variable ‘IPI’, a reference category was added representing Pulse stimuli alone (without Pre-Pulse). As a result, the interaction term ‘Genotype * IPI’ is a log odds-ratio estimating the additional effect of a given genotype on the log-odds of jumping when both Pulse and Pre-Pulse stimuli are applied (with a particular IPI) compared to the effect of the same genotype when only Pulse stimuli are applied. In other words, for a given IPI it represents the difference between two genotypes in their degree of PPI (i.e., in the effect of Pre-Pulse preceding Pulse stimuli). An odds ratio < 1 indicates a lower chance for jumping in the case of adding PPI stimulus compared to Pulse alone, implying stronger Pre-Pulse Inhibition for the genotype in the numerator.

Formula 2):

$$logit\left( p_{i} \right)= \beta_{0}+ \beta^{Genotype}\cdot{Genotype}_{i}+ \beta^{IPI}\cdot{IPI}_{i}+\beta^{Genotype\cdot IPI}\cdot{Genotype}_{i}\cdot{IPI}_{i}+$$

$$\beta^{Pulse}\cdot{Pulse}_{n[i]}+\beta^{Pulse\cdot IPI}\cdot{Pulse}_{n[i]}\cdot{IPI}_{i}+\alpha_{k[i]}^{Run}+ \alpha_{l[i]}^{Chamber}+ \alpha_{m[i]}^{Operator}+ \alpha_{n[i]}^{Fly ID}$$

, where
Pulse_n_ = ln(0.1 + relative frequency of jumping to pulse for fly*n*), e.g., if fly n jumps 8 out of 10 times, ln(0.1 + 0.8)
